# Fast covariance estimation for sparse functional data

**DOI:** 10.1007/s11222-017-9744-8

**Published:** 2017-04-11

**Authors:** Luo Xiao, Cai Li, William Checkley, Ciprian Crainiceanu

**Affiliations:** 1 Department of Statistics, North Carolina State University, Raleigh, NC USA; 2 School of Medicine, Johns Hopkins University, Baltimore, MD USA; 3 Department of Biostatistics, Johns Hopkins University, Baltimore, MD USA

**Keywords:** Bivariate smoothing, FACEs, fPCA

## Abstract

Smoothing of noisy sample covariances is an important component in functional data analysis. We propose a novel covariance smoothing method based on penalized splines and associated software. The proposed method is a bivariate spline smoother that is designed for covariance smoothing and can be used for sparse functional or longitudinal data. We propose a fast algorithm for covariance smoothing using leave-one-subject-out cross-validation. Our simulations show that the proposed method compares favorably against several commonly used methods. The method is applied to a study of child growth led by one of coauthors and to a public dataset of longitudinal CD4 counts.

**Electronic supplementary material:**

The online version of this article (doi:10.1007/s11222-017-9744-8) contains supplementary material, which is available to authorized users.

## Introduction

The covariance function is a crucial ingredient in functional data analysis. Sparse functional or longitudinal data are ubiquitous in scientific studies, while functional principal component analysis has become one of the first-line approaches to analyzing this type of data; see, e.g., Besse and Ramsay (1986), Ramsay and Dalzell (1991), Kneip (1994), Besse et al. (1997), Staniswalis and Lee (1998), Yao et al. (2003, 2005).

Given a sample of functions observed at a finite number of locations and, often, with sizable measurement error, there are usually three approaches for obtaining smooth functional principal components: (1) smooth the functional principal components of the sample covariance function; (2) smooth each curve and diagonalize the resulting sample covariance of the smoothed curves; and (3) smooth the sample covariance function and then diagonalize it.

The sample covariance function is typically noisy and difficult to interpret. Therefore, bivariate smoothing is usually employed. Local linear smoothers (Fan and Gijbels 1996), tensor-product bivariate *P*-splines (Eilers and Marx 2003) and thin plate regression splines (Wood 2003) are among the popular methods for smoothing the sample covariance function. For example, the *fpca.sc* function in the R package *refund* (Huang et al. 2015) uses the tensor-product bivariate *P*-splines. However, there are two known problems with these smoothers: (1) they are general-purpose smoothers that are not designed specifically for covariance operators; and (2) they ignore that the subject, instead of the observation, is the independent sampling unit and assume that the empirical covariance surface is the sum between an underlying smooth covariance surface and independent random noise. The FACE smoothing approach proposed by Xiao et al. (2016) was designed specifically to address these weaknesses of off-the-shelf covariance smoothing software. The method is implemented in the function *fpca.face* in the *refund* R package (Huang et al. 2015) and has proven to be reliable and fast in a range of applications. However, FACE was developed for high-dimensional dense functional data and the extension to sparse data is far from obvious. One approach that attempts to solve these problems was proposed by Yao et al. (2003). In their paper, they used leave-one-subject-out cross-validation to choose the bandwidth for local polynomial smoothing methods. This approach is theoretically sound, but computationally expensive. This may be the reason why the practice is to either try multiple bandwidths and visually inspect the results or completely ignore within-subject correlations.

Several alternative methods for covariance smoothing of sparse functional data also exist in the literature: James et al. (2000) used reduced rank spline mixed effects models, Cai and Yuan (2012) considered nonparametric covariance function under the reproducing kernel Hilbert space framework, and Peng and Paul (2009) proposed a geometric approach under the framework of marginal maximum likelihood estimation.

Our paper has two aims. First, we propose a new automatic bivariate smoother that is specifically designed for covariance function estimation and can be used for sparse functional data. Second, we propose a fast algorithm for selecting the smoothing parameter of the bivariate smoother using leave-one-subject-out cross-validation. The code for the proposed method is publicly available in the *face* R package (Xiao et al. 2017).

## Model

Suppose that the observed data take the form $\{(y_{ij}, t_{ij}), j=1,\ldots , m_i, i=1,\ldots , n\}$, where $t_{ij}$ is in the unit interval [0, 1], *n* is the number of subjects, and $m_i$ is the number of observations for subject *i*. The model is

1 $$\begin{aligned} y_{ij} = f(t_{ij}) + u_i(t_{ij}) + \varepsilon _{ij}, \end{aligned}$$

where *f* is a smooth mean function, $u_i(t)$ is generated from a zero-mean Gaussian process with covariance operator $C(s,t) = \hbox {cov}\{u_i(s), u_i(t)\}$, and $\varepsilon _{ij}$ is white noise following a normal distribution $\mathscr {N}(0, \sigma ^2_{\varepsilon })$. We assume that the random terms are independent across subjects and from each other. For longitudinal data, $m_i$’s are usually much smaller than *n*.

We are interested in estimating the covariance function $C(s,t)$. A standard procedure employed for obtaining a smooth estimate of $C(s,t)$ consists of two steps. In the first step, an empirical estimate of the covariance function is constructed. Let $r_{ij} = y_{ij} - f(t_{ij})$ be the residuals and $C_{ij_1j_2} = r_{ij_1}r_{ij_2}$ be the auxiliary variables. Because $\mathbb {E}(C_{ij_1j_2}) = C(t_{ij_1}, t_{ij_2})$ if $j_1\ne j_2$, $\{C_{ij_1j_2}: 1\le j_1\ne j_2\le m_i, i = 1,\ldots , n\}$ is a collection of unbiased empirical estimates of the covariance function. In the second step, the empirical estimates are smoothed using a bivariate smoother. Smoothing is required because the empirical estimates are usually noisy and scattered in time. Standard bivariate smoothers are local linear smoothers (Fan and Gijbels 1996), tensor-product bivariate *P*-splines (Eilers and Marx 2003) and thin plate regression splines (Wood 2003). In the following section, we propose a statistically efficient, computationally fast and automatic smoothing procedure that serves as an alternative to these approaches.

To carry out the above steps, we assume a mean function estimator $\hat{f}$ exists. Then, we let $\hat{r}_{ij} = y_{ij} - \hat{f}(t_{ij})$ and $\widehat{C}_{ij_1j_2} = \hat{r}_{ij_1}\hat{r}_{ij_2}$. Note that we use the hat notation on variables when *f* is substituted by $\hat{f}$ and when we define a variable with a hat notation, the same variable without a hat notation is similarly defined using the true *f*. In our software, we estimate *f* using a *P*-spline smoother (Eilers and Marx 1996) with the smoothing parameter selected by leave-one-subject-out cross-validation. See Section S.1 of the online supplement for details.

## Method

We model the covariance function $C(s,t)$ as a tensor-product splines $H(s,t) = \sum _{1\le \kappa \le c, 1\le \ell \le c}\theta _{\kappa \ell } B_{\kappa }(s)B_{\ell }(t)$, where ${\varvec{\varTheta }}= (\theta _{\kappa \ell })_{1\le \kappa \le c, 1\le \ell \le c}$ is a coefficient matrix, $\{B_1(\cdot ),\ldots , B_c(\cdot )\}$ is the collection of B-spline basis functions in the unit interval, and *c* is the number of interior knots plus the order (degree plus 1) of the B-splines. Note that the locations and number of knots as well as the polynomial degrees of splines determine the forms of the B-spline basis functions (de Boor 1978). We use equally spaced knots and enforce the following constraint on ${\varvec{\varTheta }}$:

$$\begin{aligned} \theta _{\kappa \ell } = \theta _{\ell \kappa },\quad 1\le \kappa ,\quad \ell \le c. \end{aligned}$$

With this constraint, *H*(*s*, *t*) is always symmetric in *s* and *t*, a desired property for estimates of covariance functions.

Unlike the other approaches covariance function estimation methods described before, our method applies a joint estimation of covariance function and error variance and incorporates the correlation structure of the auxiliary variables $\{\widehat{C}_{ij_1j_2}\,{:}\,1\le j_1\le j_2\le m_i, i = 1,\ldots , n\}$ in a two-step procedure to boost statistical efficiency. Because we use a relatively large number of knots, estimating ${\varvec{\varTheta }}$ by least squares or weighted least squares tends to overfit. Thus, we estimate ${\varvec{\varTheta }}$ by minimizing a penalized weighted least squares. Let $n_i = m_i(m_i+1)/2$, $\widehat{\mathbf{C}}_{ij} = \left\{ \widehat{C}_{i j j}, \widehat{C}_{i j (j+1)},\ldots , \widehat{C}_{i j m_i}\right\} ^T\in {\mathbb {R}}^{m_i - j + 1}$, $\mathcal {\pmb {H}}_{ij} = \{H(t_{ij}, t_{ij}), H(t_{ij}, t_{i (j+1)}), \ldots , H(t_{ij}, t_{i m_i})\}^T \in {\mathbb {R}}^{m_i - j + 1}$, and ${\varvec{\delta }}_{ij} = (1, \mathbf {0}_{m_i - j}^T)^T\in {\mathbb {R}}^{m_i - j + 1}$ for $1\le j\le m_i$. Then, let $\widehat{\mathbf{C}}_i = (\widehat{\mathbf{C}}_{i1}^T,\widehat{\mathbf{C}}_{i2}^T,\ldots , \widehat{\mathbf{C}}_{im_i}^T)^T\in {\mathbb {R}}^{n_i}$ be the vector of all auxiliary variables $\widehat{C}_{ij_1j_2}$ for subject *i* with $j_1\le j_2$. Here, $\widehat{\mathbf{C}}_i$ contains the nugget terms $\widehat{C}_{ijj}$ and note that $\mathbb {E}(C_{ijj}) = r(t_{ij}, t_{ij}) + \sigma _{\varepsilon }^2$. Similarly, we let $\mathcal {\pmb {H}}_i = (\mathcal {\pmb {H}}_{i1}^T,\mathcal {\pmb {H}}_{i2}^T,\ldots , \mathcal {\pmb {H}}_{im_i}^T)^T\in {\mathbb {R}}^{n_i}$, and ${\varvec{\delta }}_i = ({\varvec{\delta }}_{i1}^T,{\varvec{\delta }}_{i2}^T,\ldots , {\varvec{\delta }}_{im_i}^T)^T\in {\mathbb {R}}^{n_i}$. Also let $\mathbf{W}_i\in {\mathbb {R}}^{n_i\times n_i}$ be a weight matrix for capturing the correlation of $\widehat{\mathbf{C}}_i$ and will be specified later. The weighted least squares is $\hbox {WLS} = \sum _{i=1}^n \left( \mathcal {\pmb {H}}_i + {\varvec{\delta }}_i\sigma ^2_{\varepsilon } - \widehat{\mathbf{C}}_i\right) ^T\mathbf{W}_i \left( \mathcal {\pmb {H}}_i + {\varvec{\delta }}_i\sigma ^2_{\varepsilon } - \widehat{\mathbf{C}}_i\right) .$ Let $\Vert \cdot \Vert _F$ denote the Frobenius norm and let $\mathbf{D}\in {\mathbb {R}}^{c\times (c-2)}$ be a second-order differencing matrix (Eilers and Marx 1996). Then, we estimate ${\varvec{\varTheta }}$ and $\sigma _{\varepsilon }^2$ by

2 $$\begin{aligned} (\widehat{{\varvec{\varTheta }}}, \hat{\sigma }^2_{\varepsilon }) = \arg \min _{{\varvec{\varTheta }}: {\varvec{\varTheta }}= {\varvec{\varTheta }}^T, \sigma _{\varepsilon }^2} \left\{ \hbox {WLS}+\lambda \Vert {\varvec{\varTheta }}\mathbf{D}\Vert ^2_F\right\} , \end{aligned}$$

where $\lambda$ is a smoothing parameter that balances model fit and smoothness of the estimate.

The penalty term $\Vert {\varvec{\varTheta }}\mathbf{D}\Vert ^2_F$ is essentially equivalent to the penalty $\iint _{s, t} \left\{ \frac{\partial ^2 H}{\partial s^2}(s,t)\right\} ^2\mathrm {d}s\mathrm {d}t$ and can be interpreted as the row penalty in bivariate *P*-splines (Eilers and Marx 2003). Note that when ${\varvec{\varTheta }}$ is symmetric, as in our case, the row and column penalties in bivariate *P*-splines become the same. Therefore, our proposed method can be regarded as a special case of bivariate *P*-splines that is designed specifically for covariance function estimation. Another note is that when the smoothing parameter goes to infinity, the penalty term forces *H*(*s*, *t*) to become linear in both the *s* and the *t* directions. Finally, if $\widehat{\theta }_{\kappa \ell }$ denotes the $(\kappa ,\ell )$th element of $\widehat{{\varvec{\varTheta }}}$, then our estimate of the covariance function $C(s,t)$ is given by $\widetilde{C}(s,t) = \sum _{1\le \kappa \le c, 1\le \ell \le c}\widehat{\theta }_{\kappa \ell } B_{\kappa }(s)B_{\ell }(t)$.

### Estimation

Let $\mathbf{b}(t) = \{B_1(t), \ldots , B_c(t)\}^T$ be a vector. Let $\text {vec}(\cdot )$ be an operator that stacks the columns of a matrix into a vector and denote $\otimes$ the Kronecker product operator. Then $H(s,t) = \{\mathbf{b}(t)\otimes \mathbf{b}(s)\}^T\text {vec}\,{\varvec{\varTheta }}$. Let ${\varvec{\theta }}= \text {vech}\, {\varvec{\varTheta }}$, where $\text {vech}(\cdot )$ is an operator that stacks the columns of the lower triangle of a matrix into a vector, and let $\mathbf{G}_c$ be the duplication matrix (Seber 2007, p. 246) such that $\mathbf {\,}{\varvec{\varTheta }}= \mathbf{G}_c {\varvec{\theta }}$. It follows that $H(s,t) = \{\mathbf{b}(t)\otimes \mathbf{b}(s)\}^T\mathbf{G}_c {\varvec{\theta }}$.

Let $\mathbf{B}_{ij} = [\mathbf{b}(t_{ij}),\ldots , \mathbf{b}(t_{im_i})]\otimes \mathbf{b}(t_{ij})$, $\mathbf{B}_i = [\mathbf{B}_{i1}^T,\ldots , \mathbf{B}_{im_i}^T]^T$ and $\mathbf{B}= [\mathbf{B}_1^T,\ldots , \mathbf{B}_n^T]^T$. Also let $\mathbf{X}_i = [\mathbf{B}_i\mathbf{G}_c, {\varvec{\delta }}_i]$ and $\mathbf{X}= [\mathbf{X}_1^T,\ldots , \mathbf{X}_n^T]^T$. ${\varvec{\alpha }}= ({\varvec{\theta }}^T,\sigma _{\varepsilon }^2)^T$. Finally let $\widehat{\mathbf{C}}= (\widehat{\mathbf{C}}_i^T,\ldots , \widehat{\mathbf{C}}_n^T)^T$, ${\varvec{\delta }}= ({\varvec{\delta }}_1^T, \cdots , {\varvec{\delta }}_n^T)^T$ and $\mathbf{W}= \text {blockdiag}(\mathbf{W}_1,\cdots ,\mathbf{W}_n)$. Note that $\mathbf{X}$ can also be written as $[\mathbf{B}\mathbf{G}_c, {\varvec{\delta }}]$. Then,

$$\begin{aligned} \mathbb {E}(\widehat{\mathbf{C}}_i) = \mathcal {\pmb {H}}_i + {\varvec{\delta }}_i\sigma ^2_{\varepsilon } = [\mathbf{B}_i\mathbf{G}_c, {\varvec{\delta }}_i ]\begin{pmatrix} {\varvec{\theta }}^T, \delta _{\varepsilon } \end{pmatrix}^T = \mathbf{X}_i {\varvec{\alpha }}, \end{aligned}$$

and

3 $$\begin{aligned} \hbox {WLS} = \left( \widehat{\mathbf{C}}- \mathbf{X}{\varvec{\alpha }}\right) ^T \mathbf{W}\left( \widehat{\mathbf{C}}- \mathbf{X}{\varvec{\alpha }}\right) . \end{aligned}$$

Next let $\hbox {tr}(\cdot )$ be the trace operator such that for a square matrix $\mathbf{A}$, $\hbox {tr}(\mathbf{A})$ is the sum of the diagonals of $\mathbf{A}$. We can derive that (Seber 2007, p. 241)

$$\begin{aligned} \Vert {\varvec{\varTheta }}\mathbf{D}\Vert _F^2= & {} \hbox {tr}({\varvec{\varTheta }}\mathbf{D}\mathbf{D}^T{\varvec{\varTheta }}^T)\nonumber , \\= & {} (\text {vec}\,{\varvec{\varTheta }})^T (\mathbf{I}_c\otimes \mathbf{D}\mathbf{D}^T)\text {vec}\,{\varvec{\varTheta }}. \end{aligned}$$

Because $\mathbf {\,}{\varvec{\varTheta }}= \mathbf{G}_c {\varvec{\theta }}$, we obtain that

4 $$\begin{aligned} \Vert {\varvec{\varTheta }}\mathbf{D}\Vert _F^2= & {} {\varvec{\theta }}^T\mathbf{G}_c^T(\mathbf{I}_c\otimes \mathbf{D}\mathbf{D}^T)\mathbf{G}_c^T{\varvec{\theta }}\nonumber \\= & {} \begin{pmatrix} {\varvec{\theta }}^T&\sigma ^2_{\varepsilon } \end{pmatrix}\begin{pmatrix} \mathbf{P}&{} \mathbf {0} \\ \mathbf {0} &{} 0 \end{pmatrix}\begin{pmatrix} {\varvec{\theta }}\\ \sigma ^2_{\varepsilon } \end{pmatrix} \nonumber \\= & {} {\varvec{\alpha }}^T \mathbf{Q}{\varvec{\alpha }}, \end{aligned}$$

where $\mathbf{P}= \mathbf{G}_c^T(\mathbf{I}_c\otimes \mathbf{D}\mathbf{D}^T)\mathbf{G}_c^T$ and $\mathbf{Q}$ is the block matrix containing $\mathbf{P}$ and zeros.

By (3) and (4), the objective function in (2) can be rewritten as

5 $$\begin{aligned} \hat{{\varvec{\alpha }}}= \arg \min _{{\varvec{\alpha }}} \left( \widehat{\mathbf{C}}- \mathbf{X}{\varvec{\alpha }}\right) ^T \mathbf{W}\left( \widehat{\mathbf{C}}- \mathbf{X}{\varvec{\alpha }}\right) + \lambda {\varvec{\alpha }}^T \mathbf{Q}{\varvec{\alpha }}. \end{aligned}$$

Now we obtain an explicit form of $\hat{{\varvec{\alpha }}}$

6 $$\begin{aligned} \hat{{\varvec{\alpha }}} = \begin{pmatrix} \hat{{\varvec{\theta }}} \\ \hat{\sigma }^2_{\varepsilon } \end{pmatrix}= & {} \left( \mathbf{X}^T\mathbf{W}\mathbf{X}+ \lambda \mathbf{Q}\right) ^{-1}\left( \mathbf{X}^T\mathbf{W}\widehat{\mathbf{C}}\right) . \end{aligned}$$

We need to specify the weight matrices $\mathbf{W}_i$’s. One sensible choice for $\mathbf{W}_i$ is the inverse of $\hbox {cov}\left( \mathbf{C}_i\right)$, where $\mathbf{C}_i$ is defined similar to $\widehat{\mathbf{C}}_i$, except that the true mean function *f* is used. However, $\hbox {cov}\left( \mathbf{C}_i\right)$ may not be invertible or may be close to being singular. Thus, we specify $\mathbf{W}_i$ as

$$\begin{aligned} \mathbf{W}_i^{-1}= & {} (1-\beta ) \hbox {cov}\left( \mathbf{C}_i\right) +\beta \text {diag}\left\{ \text {diag}\left\{ \hbox {cov}\left( \mathbf{C}_i\right) \right\} \right\} ,\\&1\le i\le n, \end{aligned}$$

for some constant $0<\beta < 1$. The above specification ensures that $\mathbf{W}_i$ exists and is stable. We will use $\beta = 0.05$, which works well in practice.

We now derive $\hbox {cov}\left( \mathbf{C}_i\right)$ in terms of $C$ and $\sigma _{\varepsilon }^2$. First note that $\mathbb {E}(r_{ij_1}r_{ij_2}) = \hbox {cov}(r_{ij_1},r_{ij_2}) = C(t_{ij_1},t_{ij_2}) + \delta _{j_1j_2}\sigma ^2_{\varepsilon },$ where $\delta _{j_1j_2} = 1$ if $j_1 = j_2$ and 0 otherwise.

#### Proposition 1

Define $$\mathbf{M}_{ijk} = \left\{ C(t_{ij},t_{ik}), \delta _{jk}\sigma ^2_{\varepsilon }\right\} ^T \in {\mathbb {R}}^2$$. Then,

$$\begin{aligned} \hbox {cov}\left( C_{ij_1j_2},C_{ij_3j_4}\right) = \mathbf {1}^T(\mathbf{M}_{ij_1j_3} \otimes \mathbf{M}_{ij_2j_4} +\mathbf{M}_{ij_1j_4} \otimes \mathbf{M}_{ij_2j_3}). \end{aligned}$$

The proof of Proposition 1 is provided in Section S.2 of the online supplement. Now we see that $\mathbf{W}_i$ also depends on $(C,\sigma ^2_{\varepsilon })$. Hence, we employ a two-stage estimation. We first estimate $(C,\sigma ^2_{\varepsilon })$ by using penalized ordinary least squares, i.e., $\mathbf{W}_i = \mathbf{I}$ for all *i*. Then, we obtain the plug-in estimate of $\mathbf{W}_i$ and estimate $(C,\sigma ^2_{\varepsilon })$ using penalized weighted least squares. The algorithm for the two-stage estimation is summarized as Algorithm 1.
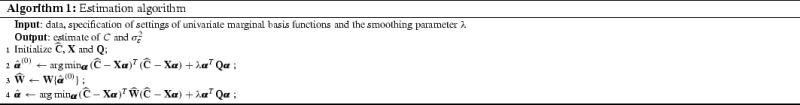


### Selection of the smoothing parameter

For selecting the smoothing parameter, we use leave-one-subject-out cross-validation, a popular approach for correlated data; see, for example, Yao et al. (2003), Reiss et al. (2010) and Xiao et al. (2015). Compared to the leave-one-observation-out cross-validation, which ignores the correlation, leave-one-subject-out cross-validation was reported to be more robust against overfit. However, such an approach is usually computationally expensive. In this section, we derive a fast algorithm for approximating the leave-one-subject-out cross-validation.

Let $\widetilde{\mathbf{C}}_i^{[i]}$ be the prediction of $\widehat{\mathbf{C}}_i$ by applying the proposed method to the data without the data from the *i*th subject, and then the cross-validated error is

7 $$\begin{aligned} \text {iCV} = \sum _{i=1}^n \Vert \widetilde{\mathbf{C}}_i^{[i]} - \widehat{\mathbf{C}}_i\Vert ^2. \end{aligned}$$

There is a simple formula for iCV. First, we let $\mathbf{S}= \mathbf{X}(\mathbf{X}^T\mathbf{W}\mathbf{X}+ \lambda \mathbf{Q})^{-1}\mathbf{X}^T\mathbf{W}$, which is the smoother matrix for the proposed method. $\mathbf{S}$ can be written as $(\mathbf{X}\mathbf{A})[\mathbf{I}+ \lambda \hbox {diag}(\mathbf{s})]^{-1}(\mathbf{X}\mathbf{A})^T\mathbf{W}$ for some square matrix $\mathbf{A}$ and $\mathbf{s}$ is a column vector; see, for example, Xiao et al. (2013). In particular, both $\mathbf{A}$ and $\mathbf{s}$ do not depend on $\lambda$.

Let $\mathbf{S}_i = \mathbf{X}_i(\mathbf{X}^T\mathbf{W}\mathbf{X}+ \lambda \mathbf{Q})^{-1}\mathbf{X}^T\mathbf{W}$ and $\mathbf{S}_{ii} = \mathbf{X}_i(\mathbf{X}^T\mathbf{W}\mathbf{X} + \lambda \mathbf{Q})^{-1}\mathbf{X}_i^T\mathbf{W}_i$. Then, $\mathbf{S}_i$ is of dimension $n_i\times N$, where $N = \sum _{i=1}^n n_i$, and $\mathbf{S}_{ii}$ is of dimension $n_i\times n_i$.

#### Lemma 1

The ${\mathrm{iCV}}$ in (7) can be simplified as

$$\begin{aligned} {\mathrm{iCV}} = \sum _{i=1}^n \Vert (\mathbf{I}_{n_i}-\mathbf{S}_{ii})^{-1}(\mathbf{S}_i\widehat{\mathbf{C}}- \widehat{\mathbf{C}}_i)\Vert ^2. \end{aligned}$$

The proof of Lemma 1 is the same as that of Lemma 3.1 in Xu and Huang (2012) and thus is omitted. Similar to Xu and Huang (2012), we further simplify iCV by using the approximation $(\mathbf{I}_{n_i}-\mathbf{S}_{ii}^T)^{-1}(\mathbf{I}_{n_i}-\mathbf{S}_{ii})^{-1} = \mathbf{I}_{n_i} + \mathbf{S}_{ii} + \mathbf{S}_{ii}^T$. This approximation leads to the generalized cross-validation, which we denote as iGCV,

8 $$\begin{aligned} \text {iGCV}= & {} \sum _{i=1}^n (\mathbf{S}_i\widehat{\mathbf{C}}- \widehat{\mathbf{C}}_i)^T (\mathbf{I}_{n_i}+\mathbf{S}_{ii} + \mathbf{S}_{ii}^T)(\mathbf{S}_i\widehat{\mathbf{C}}- \widehat{\mathbf{C}}_i)\nonumber \\= & {} \Vert \widehat{\mathbf{C}}-\mathbf{S}\widehat{\mathbf{C}}\Vert ^2 + 2\sum _{i=1}^n \left( \mathbf{S}_i\widehat{\mathbf{C}}- \widehat{\mathbf{C}}_i\right) ^T \nonumber \\&\mathbf{S}_{ii}\left( \mathbf{S}_i\widehat{\mathbf{C}}- \widehat{\mathbf{C}}_i\right) . \end{aligned}$$

While iGCV in (8) is much easier to compute than iCV in (7), the formula in (8) is still computationally expensive as the smoother matrix $\mathbf{S}$ is of dimension $N\times N$, where $N=2,000$ if $n=100$ and $m_i = m =5$ for all *i*. Thus, we further simplify iGCV.

Let $\mathbf{F}_i = \mathbf{X}_i\mathbf{A}$, $\mathbf{F}= \mathbf{X}\mathbf{A}$ and $\widetilde{\mathbf{F}}= \mathbf{F}^T\mathbf{W}$. Define $\pmb f_i = \mathbf{F}_i^T\widehat{\mathbf{C}}_i$, $\pmb f= \mathbf{F}^T\widehat{\mathbf{C}}$, $\widetilde{\mathbf{f}}= \widetilde{\mathbf{F}}\widehat{\mathbf{C}}$, $\mathbf{J}_i = \mathbf{F}_i^T\mathbf{W}_i\widehat{\mathbf{C}}_i$, $\mathbf{L}_i = \mathbf{F}_i^T\mathbf{F}_i$ and $\widetilde{\mathbf{L}}_i = \mathbf{F}_i^T\mathbf{W}_i\mathbf{F}_i$. To simplify notation we will denote $[\mathbf{I}+ \lambda \hbox {diag}(\mathbf{s})]^{-1}$ as $\widetilde{\mathbf{D}}$, a symmetric matrix, and its diagonal as $\widetilde{\mathbf{d}}$. Let $\odot$ be the Hadamard product such that for two matrices of the same dimensions $A = (a_{ij})$ and $B=(b_{ij})$, $A\odot B = (a_{ij}b_{ij})$.

#### Proposition 2

The $\text {iGCV}$ in (8) can be simplified as

$$\begin{aligned} \text {iGCV}= & {} \Vert \widehat{\mathbf{C}}\Vert ^2 - 2\widetilde{\mathbf{d}}^T(\widetilde{\mathbf{f}}\odot \pmb f) + (\widetilde{\mathbf{f}}\odot \widetilde{\mathbf{d}})^T(\mathbf{F}^T\mathbf{F})(\widetilde{\mathbf{f}}\odot \widetilde{\mathbf{d}}) \nonumber \\&+\,2 \widetilde{\mathbf{d}}^T\pmb g- 4\widetilde{\mathbf{d}}^T \mathbf{G}\widetilde{\mathbf{d}}+ 2 \widetilde{\mathbf{d}}^T\nonumber \\&\left[ \sum _{i=1}^n\left\{ \mathbf{L}_i(\widetilde{\mathbf{f}}\odot \widetilde{\mathbf{d}})\right\} \odot \left\{ \widetilde{\mathbf{L}}_i(\widetilde{\mathbf{f}}\odot \widetilde{\mathbf{d}})\right\} \right] , \end{aligned}$$

where $$\pmb g= \sum _{i=1}^n \mathbf{J}_i\odot \pmb f_i$$ and $$\mathbf{G}= \sum _{i=1}^n(\mathbf{J}_i{\widetilde{\mathbf{f}}}^T)\odot \mathbf{L}_i$$.

The proof of Proposition 2 is provided in Section S.2 of the online supplement.
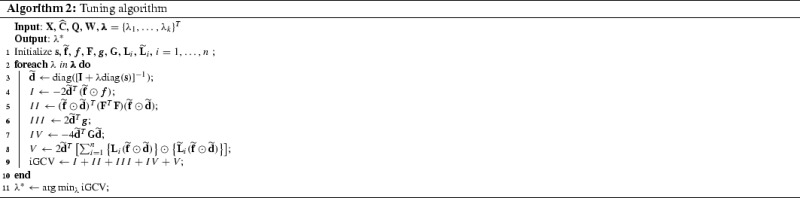


While the formula in Proposition 2 looks complex, it can be efficiently computed. Indeed, only the term $\widetilde{\mathbf{d}}$ depends on the smoothing parameter $\lambda$ and it can be easily computed; all other terms including $\pmb g$ and $\mathbf{G}$ can be pre-calculated just once. Suppose the number of observations per subject is $m_i = m$ for all *i*. Let $K= c(c+1)/2 + 1$ and $M= m(m+1)/2$. Note that *K* is the number of unknown coefficients and *M* is the number of raw covariances from each subject. Then, the pre-calculation of terms in the iGCV formula requires $O(nMK^2 +nM^2K + K^3 + M^3)$ computation time and each calculation of iGCV requires $O(nK^2)$ computation time. To see the efficiency of the simplified formula in Proposition 2, we note that a brute force evaluation of iCV in Lemma 1 requires computation time of the order $O(nM^3 + nK^3 + n^2M^2K)$, quadratic in the number of subjects *n*.

When the number of observations per subject *m* is small, i.e., $m < c$, the number of univariate basis functions, the iGCV computation time increases linearly with respect to *m*; when *m* is relatively large, i.e., $m>c$ but $m = o(n)$, the iGCV computation time increases quadratically with respect to *m*. Therefore, the iGCV formula is most efficient with a small *m*, i.e., sparse data. As for the case that *m* is very large and the proposed method becomes very slow, the method in Xiao et al. (2016) might be preferred.

## Curve prediction

In this section, we consider the prediction of $X_i(t) = f(t) + u_i(t)$, the *i*th subject curve. We assume that $X_i(t)$ is generated from a Gaussian process. Suppose we would like to predict $X_i(t)$ at $\{s_{i1},\ldots , s_{im}\}$ for $m\ge 1$. Let $\mathbf{y}_i = (\mathbf{y}_{i1},\ldots ,\mathbf{y}_{im_i})^T$, $\pmb f_i^o = \{f(t_{i1}),\ldots , f(t_{im_i})\}^T$, and $\mathbf {x}_i = \{X_i(s_{i1}),\ldots , X_i(s_{im})\}^T$. Let $\mathbf{H}_i^o = [\mathbf{b}(t_{i1}),\ldots , \mathbf{b}(t_{im_i})]^T$ and $\mathbf{H}_i^n = [\mathbf{b}(s_{i1}),\ldots , \mathbf{b}(s_{im})]^T$. It follows that

$$\begin{aligned} \left( \begin{array}{c}\mathbf{y}_i \\ \mathbf {x}_i\end{array}\right)\sim & {} \mathscr {N}\left\{ \left( \begin{array}{c}\pmb f_i^o \\ \pmb f_i^n\end{array}\right) , \left( \begin{array}{cc}\mathbf{H}_i^o{\varvec{\varTheta }}\mathbf{H}_i^{o,T}&{} \mathbf{H}_i^o{\varvec{\varTheta }}\mathbf{H}_i^{n,T} \\ \mathbf{H}_i^n{\varvec{\varTheta }}\mathbf{H}_i^{o,T} &{} \mathbf{H}_i^n{\varvec{\varTheta }}\mathbf{H}_i^{n,T} \end{array}\right) \right. \\&\left. +\,\sigma _{\varepsilon }^2\mathbf{I}_{m_i + m} \right\} . \end{aligned}$$

We derive that

$$\begin{aligned} \mathbb {E}(\mathbf {x}_i|\mathbf{y}_i) = \left( \mathbf{H}_i^n{\varvec{\varTheta }}\mathbf{H}_i^{o,T} \right) \mathbf{V}_i^{-1} (\mathbf{y}_i - \pmb f_i^o) + \pmb f_i^n, \end{aligned}$$

where $$\mathbf{V}_i = \mathbf{H}_i^o{\varvec{\varTheta }}\mathbf{H}_i^{o,T} + \sigma _{\varepsilon }^2\mathbf{I}_{m_i} $$, and

$$\begin{aligned} \hbox {cov}(\mathbf {x}_i|\mathbf{y}_i) = \mathbf{V}_i^n -\left( \mathbf{H}_i^n{\varvec{\varTheta }}\mathbf{H}_i^{o,T} \right) \mathbf{V}_i^{-1} \left( \mathbf{H}_i^n{\varvec{\varTheta }}\mathbf{H}_i^{o,T} \right) ^T, \end{aligned}$$

where $\mathbf{V}_i^n = \mathbf{H}_i^n{\varvec{\varTheta }}\mathbf{H}_i^{n,T} + \sigma _{\varepsilon }^2\mathbf{I}_{m}$. Because *f*, ${\varvec{\varTheta }}$ and $\sigma _{\varepsilon }^2$ are unknown, we need to plug in their estimates $\hat{f}$, $\widehat{{\varvec{\varTheta }}}$ and $\hat{\sigma }^2_{\varepsilon }$, respectively, into the above equalities. Thus, we could predict $\mathbf {x}_i$ by

$$\begin{aligned} \hat{\mathbf {x}}_i= & {} \{\hat{x}_i(s_{i1}),\ldots , \hat{x}_i(s_{im})\}^T \\= & {} \left( \mathbf{H}_i^n\widehat{{\varvec{\varTheta }}}\mathbf{H}_i^{o,T} \right) \hat{\mathbf{V}}_i^{-1} (\mathbf{y}_i - \hat{\pmb f_i^o}) + \hat{\pmb f_i^n}, \end{aligned}$$

where $\hat{\pmb f_i^o}= \{\hat{f}(t_{i1}),\ldots , \hat{f}(t_{im_i})\}^T$, $\hat{\pmb f_i^n} = \{\hat{f}(s_{i1}),\ldots , \hat{f}(s_{im})\}^T$, and $\hat{\mathbf{V}}_i = \mathbf{H}_i^o\hat{{\varvec{\varTheta }}}\mathbf{H}_i^{o,T} + \hat{\sigma }_{\varepsilon }^2\mathbf{I}_{m_i}$. Moreover, an approximate covariance matrix for $\hat{\mathbf {x}}_i$ is

$$\begin{aligned} \widehat{\hbox {cov}}(\hat{\mathbf {x}}_i |\mathbf{y}_i) = \hat{\mathbf{V}}_i^n -\left( \mathbf{H}_i^n\hat{{\varvec{\varTheta }}}\mathbf{H}_i^{o,T} \right) \hat{\mathbf{V}}_i^{-1} \left( \mathbf{H}_i^n\hat{{\varvec{\varTheta }}}\mathbf{H}_i^{o,T} \right) ^T, \end{aligned}$$

where $$\hat{\mathbf{V}}^n_i = \mathbf{H}_i^n\widehat{{\varvec{\varTheta }}}\mathbf{H}_i^{n,T} + \hat{\sigma }_{\varepsilon }^2\mathbf{I}_{m} $$.

Note that one may also use the standard Karhunen–Loeve decomposition representation of $X_i(t)$ for prediction; see, e.g., Yao et al. (2005). An advantage of the above formulation is that we avoid the evaluation of the eigenfunctions extracted from the covariance function $C$; indeed, we just need to compute the B-spline basis functions at the desired time points, which is computationally simple.

## Simulations

### Simulation setting

We generate data using model (1). The number of observations for each random curve is generated from a uniform distribution on either $\{3,4,5,6,7\}$ or $\{j: 5\le j\le 15\}$, and then observations are sampled from a uniform distribution in the unit interval. Therefore, on average, each curve has $m=5$ or $m=10$ observations. The mean function is $\mu (t) = 5\sin (2\pi t)$. For the covariance function $C(s,t)$, we consider two cases. For case 1, we let $C_1(s,t) = \sum _{\ell =1}^3 \lambda _{\ell } \psi _{\ell }(s)\psi _{\ell }(t)$, where $\psi _{\ell }$’s are eigenfunctions and $\lambda _{\ell }$’s are eigenvalues. Here, $\lambda _{\ell } = 0.5^{\ell -1}$ for $\ell =1, 2, 3$ and $\psi _1(t) = \sqrt{2}\sin (2\pi t)$, $\psi _2(t) = \sqrt{2}\cos (4\pi t)$ and $\psi _3(t) = \sqrt{2}\sin (4\pi t)$. For case 2, we consider the Matern covariance function

$$\begin{aligned} C(d;\phi ,\nu ) = \frac{1}{2^{\nu -1}\varGamma (\nu )}\left( \frac{\sqrt{2\nu }d}{\phi }\right) ^{\nu } K_{\nu }\left( \frac{\sqrt{2\nu }d}{\phi }\right) \end{aligned}$$

with range $\phi = 0.07$ and order $\nu =1$. Here, $K_{\nu }$ is the modified Bessel function of order $\nu$. The top two eigenvalues for this covariance function are 0.209 and 0.179, respectively. The noise term $\varepsilon _{ij}$’s are assumed normal with mean zero and variance $\sigma _{\varepsilon }^2$. We consider two levels of signal to noise ratio (SNR): 2 and 5. For example, if

$$\begin{aligned} \sigma ^2_{\varepsilon } = \frac{1}{2}\int _{s=0}^1 \int _{t=0}^1 C(s,t)\mathrm {d}s\mathrm {d}t, \end{aligned}$$

then the signal-to-noise ratio in the data is 2. The number of curves is $n=100$ or 400 and for each covariance function 200 datasets are drawn. Therefore, we have 16 different model conditions to examine.

### Competing methods and evaluation criterion

We compare the proposed method (denoted by FACEs) with the following methods: (1) The *fpca.sc* method in Goldsmith et al. (2010), which uses tensor-product bivariate *P*-splines (Eilers and Marx 2003) for covariance smoothing and is implemented in the R package *refund*; (2) a variant of *fpca.sc* that uses thin plate regression splines for covariance smoothing, denoted by TPRS, and is coded by the authors; 3) the MLE method in Peng and Paul (2009), implemented in the R package *fpca*; and 4) the local polynomial method in Yao et al. (2003), denoted by *loc*, and is implemented in the MATLAB toolbox *PACE*. The underlying covariance smoothing R function for *fpca.sc* and TPRS is *gam* in the R package *mgcv* (Wood 2013). For FACEs, we use $c=10$ marginal cubic B-spline bases in each dimension. To evaluate the effect of the weight matrices in the proposed objective function (2), we also report results of FACEs without using weight matrices; we denote the one stage fit by FACEs (1-stage). For *fpca.sc*, we use its default setting, which uses 10 B-spline bases in each dimension and the smoothing parameters are selected by “REML.” We also code *fpca.sc* ourselves because the *fpca.sc* function in the *refund* R package incorporates other functionalities and may become very slow. For TPRS, we also use the default setting in *gam*, with the smoothing parameter selected by “REML.” For bivariate smoothing, the default TPRS uses 27 nonlinear basis functions, in addition to the linear basis functions. We also consider TPRS with 97 nonlinear basis functions to match the basis dimension used in *fpca.sc* and FACEs. For the method MLE, we specify the range for the number of B-spline bases to be [6, 10] and the range of possible ranks to be [2, 6]. We will not evaluate the method using a reduced rank mixed effects model (James et al. 2000) because it has been shown in Peng and Paul (2009) that the MLE method is more superior.

We evaluate the above methods using four criterions. The first is the integrated squared errors (ISE) for estimating the covariance function. The next two criterions are based on the eigendecomposition of the covariance function: $C(s,t) = \sum _{\ell =1}^{\infty } \lambda _{\ell }\psi _{\ell }(s)\psi _{\ell }(t)$, where $\lambda _1 \ge \lambda _2 \ge \ldots$ are eigenvalues and $\psi _1(t),\psi _2(t),\ldots$ are the associated orthonormal eigenfunctions. The second criterion is the integrated squared errors (ISE) for estimating the top 3 eigenfunctions from the covariance function. Let $\psi (t)$ be the true eigenfunction and $\hat{\psi }(t)$ be an estimate of $\psi (t)$, then the integrated squared error is

$$\begin{aligned} \min \left[ \int _{t=0}^1 \{\psi (t) - \hat{\psi }(t)\}^2 \mathrm {d}t, \int _{t=0}^1 \{\psi (t) + \hat{\psi }(t)\}^2 \mathrm {d}t\right] . \end{aligned}$$

It is easy to show that the range of integrated squared error for eigenfunction estimation is [0, 2]. Note that for the method MLE, if rank 2 is selected, then only two eigenfunctions can be extracted. In this case, to evaluate accuracy of estimating the third eigenfunction, we will let ISE be 1 for a fair comparison. The third criterion is the squared errors (SE) for estimating the top 3 eigenvalues. The last criterion is the methods’ computation speed.

**Fig. 1:**
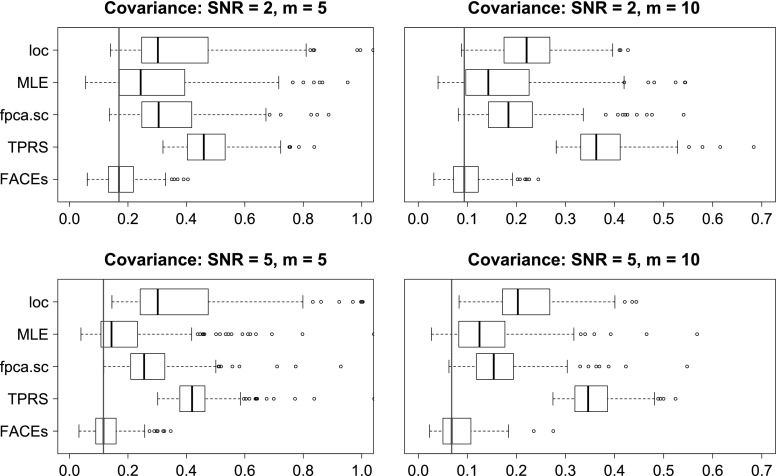
Boxplots of ISEs of five estimators for estimating the covariance functions of case 1, $n=100$

**Fig. 2:**
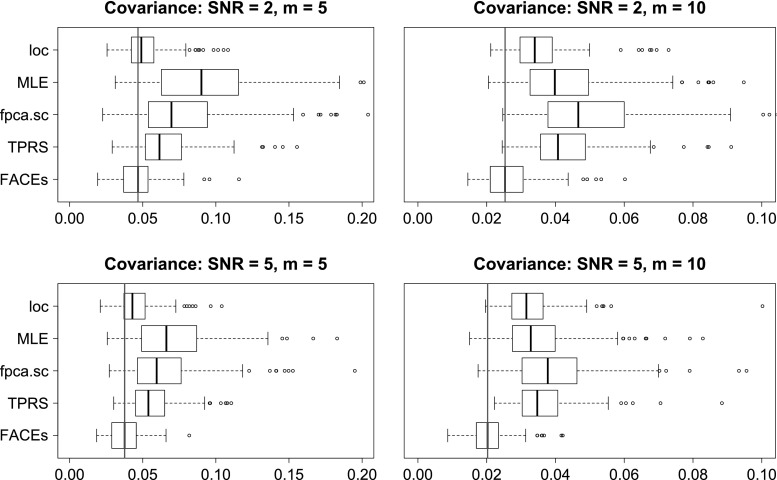
Boxplots of ISEs of five estimators for estimating the covariance functions of case 2, $n=100$

**Fig. 3:**
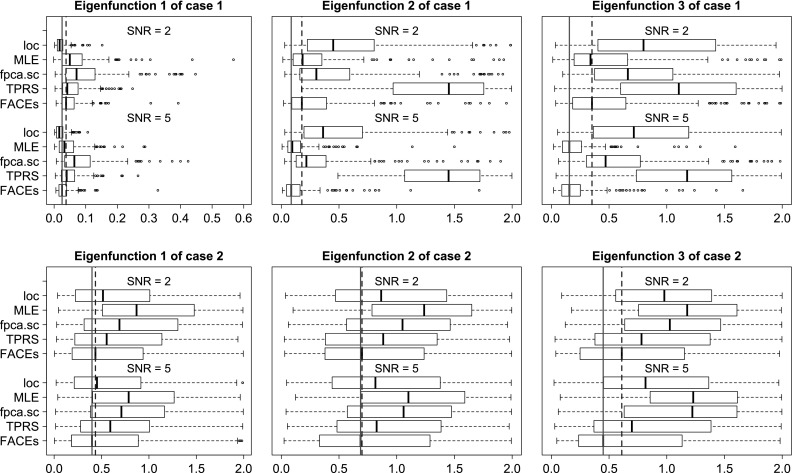
Boxplots of ISEs of five estimators for estimating the top 3 eigenfunctions when $n=100, m=5$. Note that the *straight lines* are the medians of FACEs when $\hbox {SNR} = 5$ and the *dash lines* are the medians of FACEs when $\hbox {SNR} = 2$

**Fig. 4:**
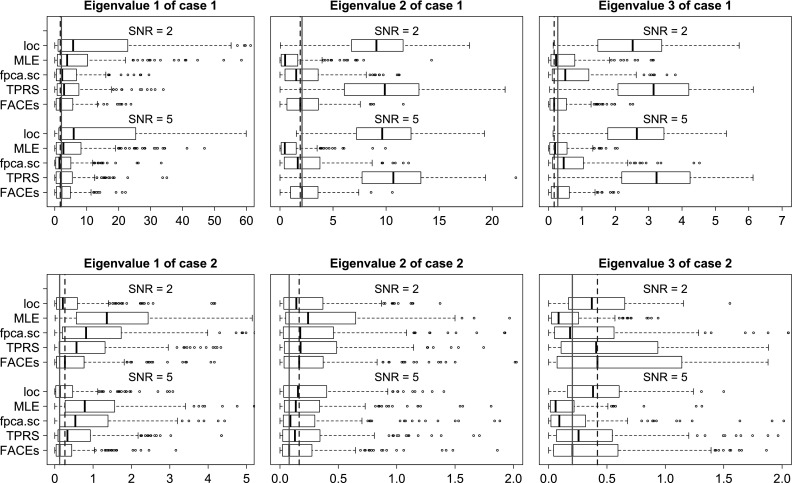
Boxplots of $100\times$ SEs of five estimators for estimating the eigenvalues when $n=100, m=5$. Note that the *straight lines* are the medians of FACEs when $\hbox {SNR} = 5$ and the *dash lines* are the medians of FACEs when $\hbox {SNR} = 2$

**Fig. 5:**
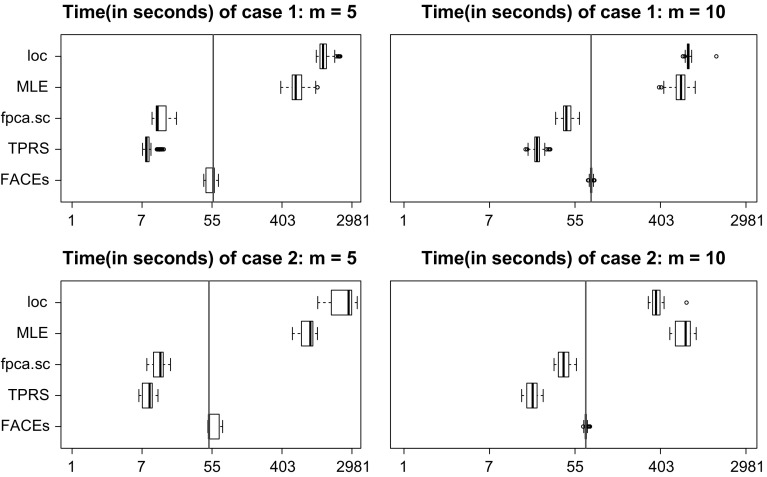
Boxplots of computation times (in seconds) of five estimators for estimating the covariance functions when $n=400, \hbox {SNR}=2$. Note that the *x*-axis is not equally spaced

**Table 1:** Median and IQR (in parenthesis) of ISEs for curve fitting for case 1

*n*	*m*	SNR	FACEs	*FAMM*(FACEs)	*fpca.sc*	*FAMM*(*fpca.sc*)	*loc*	*pffr*
100	5	2	0.714 (0.085)	**0.699** (0.102)	0.790 (0.156)	0.765 (0.147)	0.826 (0.135)	1.178 (0.092)
400	5	2	**0.592** (0.058)	0.596 (0.058)	0.625 (0.077)	0.639 (0.076)	0.735 (0.082)	1.181 (0.093)
100	10	2	0.369 (0.047)	**0.355** (0.044)	0.420 (0.066)	0.405 (0.069)	0.456 (0.076)	0.880 (0.060)
400	10	2	0.323 (0.027)	**0.317** (0.031)	0.330 (0.036)	0.336 (0.035)	0.406 (0.042)	0.872 (0.065)
100	5	5	0.497 (0.074)	**0.476** (0.082)	0.617 (0.171)	0.585 (0.147)	0.636 (0.106)	1.080 (0.109)
400	5	5	0.375 (0.042)	**0.372** (0.042)	0.416 (0.060)	0.419 (0.055)	0.523 (0.066)	1.050 (0.101)
100	10	5	0.218 (0.044)	**0.202** (0.040)	0.259 (0.056)	0.246 (0.053)	0.294 (0.058)	0.734 (0.071)
400	10	5	0.164 (0.019)	**0.160** (0.021)	0.182 (0.028)	0.180 (0.026)	0.243 (0.034)	0.740 (0.066)

The results are based on 200 replications. Numbers in boldface are the smallest of each row

### Simulation results

The detailed simulation results are presented in Section S.3 of the online supplement. Here, we provide summaries of the results along with some illustrations. In terms of estimating the covariance function, for most model conditions, FACEs gives the smallest medians of integrated squared errors and has the smallest inter-quarter ranges (IQRs). MLE is the 2nd best for case 1, while *loc* is the 2nd best for case 2. See Figs. 1 and 2 for illustrations under some model conditions.

In terms of estimating the eigenfunctions, FACEs tends to outperform other approaches in most scenarios, while for the remaining scenarios, its performance is still comparable with the best one. MLE performs well for case 1 but relatively poorly for case 2, while the opposite is true for *loc*. TPRS and *fpca.sc* perform quite poorly for estimating the 2nd and 3rd eigenfunctions in both case 1 and case 2. Figure 3 illustrates the superiority of FACEs for estimating eigenfunctions when $n=100, m=5$.

As for estimation of eigenvalues, we have the following findings: (1) FACEs performs the best for estimating the first eigenvalue in case 1; (2) *loc* performs the best for estimating the first eigenvalue in case 2; (3) MLE performs overall the best for estimating 2nd and 3rd eigenvalues in both cases, while the performance of FACEs is very close and can be better than MLE under some model scenarios; (4) TPRS, *fpca.sc* and *loc* perform quite poorly for estimating the 2nd and 3rd eigenvalues in most scenarios. We conclude that FACEs shows overall very competitive performance and never deviates much from the best performance. Figure 4 illustrates the patterns of eigenvalue estimation for $n=100, m=5$.

We now compare run times of the various methods; see Fig. 5 for an illustration. When $m=5$, FACEs takes about four to seven times the computation times of TPRS and *fpca.sc*; but it is much faster than MLE and *loc*, the speed-up is about 15 and 35 folds, respectively. When $m=10$, although FACEs is still slower than TPRS and *fpca.sc*, the computation times are similar; computation times of MLE and *loc* are over 9 and 10 folds of FACEs, respectively. Because TPRS and *fpca.sc* are naive covariance smoothers, their fast speed is offset by their tendency to have inferior performance in terms of estimation of covariance functions, eigenfunctions, and eigenvalues.

Finally, by comparing results of FACEs with its 1-stage counterpart (see the online supplement), we see that taking into account of the correlations in the raw covariances boosts the estimation accuracies of FACEs a lot. The 1-stage FACEs is of course faster. It is interesting to note that the 1-stage FACEs is actually also very competitive against other methods.

To summarize, FACEs is a relatively fast method coupled with competing performance against the methods examined above.

### Additional simulations for curve prediction

We conduct additional simulations to evaluate the performance of the FACEs method for curve prediction. We focus on case 1 and use the same simulation settings in Sect. 5.1 for generating the training data and the testing data. We generate 200 new subjects for testing. The number of observations for the subjects are generated in the same way as the training data.

In addition to the conditional expectation approach outlined in Sect. 4, Cederbaum et al. (2016) proposed a new prediction approach (denoted by FAMM). As functional data have a mixed effects representation conditional on eigenfunctions, the standard prediction procedure for mixed effects models can be used for curve prediction. The FAMM requires estimates of eigenfunctions and is applicable to any covariance smoothing method. Finally, direct estimation of subject-specific curves has also been proposed in the literature (Durban et al. 2005; Chen and Wang 2011; Scheipl et al. 2015).

We will compare the following methods: (1) the conditional expectation method using FACEs; (2) the conditional expectation method using *fpca.sc*; (3) the conditional FAMM method using FACEs; (4) the conditional FAMM method using *fpca.sc*; (5) the conditional expectation method using *loc*; and (6) the spline-based approach in Scheipl et al. (2015) without estimating covariance function, denoted by *pffr*, and is implemented in the R package *refund*. This method uses direct estimation of subject-specific curves. For the conditional FAMM approach, we follow Cederbaum et al. (2016) and fix smoothing parameters at the ratios of the estimated eigenvalues and error variance from covariance function. Fixing smoothing parameters significantly reduces the computation times of the FAMM approach.

We evaluate the above methods using the integrated squared errors, and the results are summarized in Table 1. The results show that either approach (conditional expectation or conditional FAMM) using FACEs has overall smaller prediction errors than competing approaches. The conditional FAMM approach using FACEs is slightly better than the conditional expectation approach. The results suggest that better estimation of the covariance function leads to more accurate prediction of subject-specific curves.

## Applications

We illustrate the proposed method on a publicly available dataset. Another application on a child growth dataset is provided in Section S.4 of the online supplement.

**Fig. 6:**
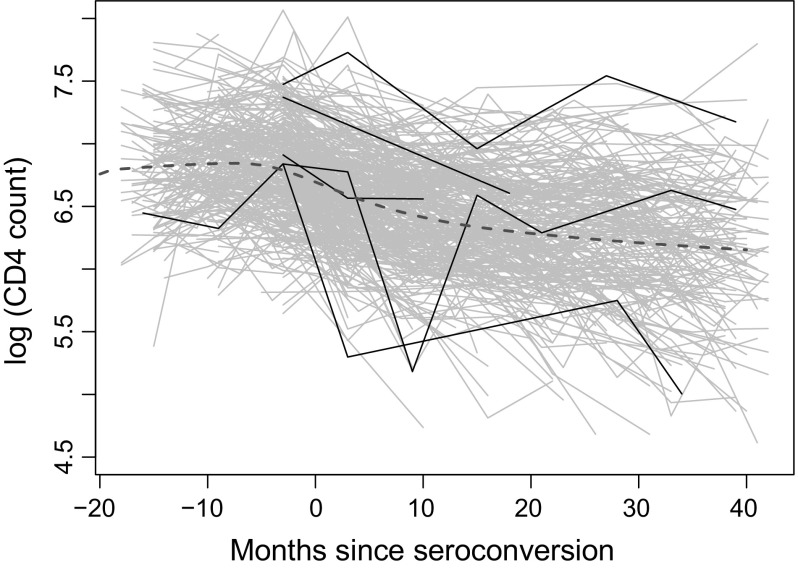
Observed $\log$ (CD4 count) trajectories of 366 HIV-infected males. The estimated population mean is the *black solid line*

**Fig. 7:**
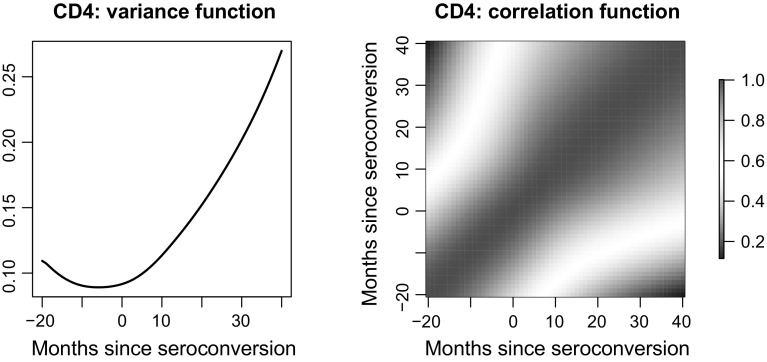
Estimated variance function (*left panel*) and correlation function (*right panel*) for the $\log$ (CD4 count)

**Fig. 8:**
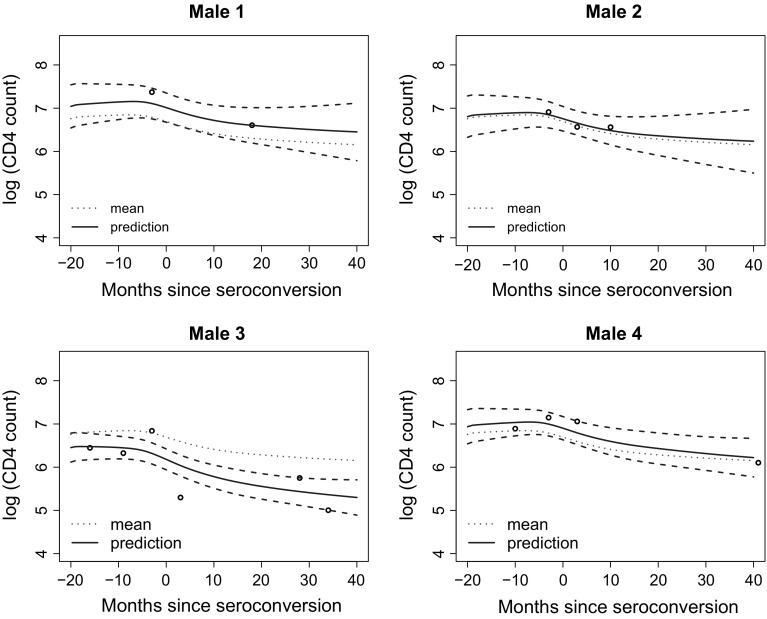
Predicted subject-specific trajectories of $\log$ (CD4 count) and associated 95% confidence bands for 4 males. The estimated population mean is the *dotted line*

CD4 cells are a type of white blood cells that could send signals to the human body to activate the immune response when they detect viruses or bacteria. Thus, the CD4 count is an important biomarker used for assessing the health of HIV-infected persons as HIV viruses attack and destroy the CD4 cells. The dataset analyzed here is from the Multicenter AIDS Cohort Study (MACS) and is available in the *refund* R package (Huang et al. 2015). The observations are CD4 cell counts for 366 infected males in a longitudinal study (Kaslow et al. 1987). With a total of 1888 data points, each subject has between 1 and 11 observations. Statistical analysis based on this or related datasets were done in Diggle et al. (1994), Yao et al. (2005), Peng and Paul (2009) and Goldsmith et al. (2013).

For our analysis, we consider $\log$ (CD4 count) since the counts are skewed. We plot the data in Fig. 6 where the *x*-axis is months since seroconversion (i.e., the time at which HIV becomes detectable). The overall trend seems to be decreasing, as can be visually confirmed by the estimated mean function plotted in Fig. 6. The estimated variance and correlation functions are displayed in Fig. 7. It is interesting to see that the minimal value of the estimated variance function occurs at month 0 since seroconversion. Finally, we display in Fig. 8 the predicted trajectory of $\log$ (CD4 count) for 4 males and the corresponding pointwise confidence bands.

## Discussion

Estimating and smoothing covariance operators is an old problem with many proposed solutions. Automatic and fast covariance smoothing is not fully developed, and, in practice, one still does not have a method that is used consistently. The reason why the practical solution to the problem has been quite elusive is the lack of automatic covariance smoothing software. The novelty of our proposal is that it directly tackles this problem from the point of view of practicality. Here, we proposed a method that we are already using extensively in practice and which is becoming increasingly popular among practitioners.

The ingredients of the proposed approach are not all new, but their combination leads to a complete product that can be used in practice. The fundamentally novel contributions that make everything practical are: (1) use a particular type of penalty that respects the covariance matrix format; (2) provide a very fast fitting algorithm for leave-one-subject-out cross-validation; and (3) ensure the scalability of the approach by controlling the overall complexity of the algorithm.

Smoothing parameters are an important component in smoothing and usually selected by either cross-validation or likelihood-based approaches. The latter make use of the mixed model representation of spline-based smoothing (Ruppert et al. 2003) and tend to perform better than cross-validation (Reiss and Todd Ogden 2009; Wood 2011). New optimization techniques have been developed (Rodríguez-Álvarez et al. 2015, 2016; Wood and Fasiolo 2017) for likelihood-based approaches. Likelihood-based approaches seem impractical for smoothing of raw covariances because the entries are products of normal residuals. Moreover, the raw covariances are dependent within subjects, which imposes additional challenge. Developing likelihood-based selection of smoothing parameters for covariance smoothing is of interest but beyond the scope of the paper.

To make methods transparent and reproducible, the method has been made publicly available in the *face* package and will be incorporated in the function *fpca.face* in the *refund* package later. The current *fpca.face* function (Xiao et al. 2016) deals with high-dimensional functional data observed on the same grid and has been used extensively by our collaborators. We have a long track-record of releasing functional data analysis software and the final form of the function will be part of the next release of *refund*.

## Electronic supplementary material

Below is the link to the electronic supplementary material.

Supplementary material 1 (pdf 702 KB)

Supplementary material 2 (gz 14 KB)
