# Supplementary material for: Fast covariance estimation for sparse functional data
Source: Stat Comput. 2017 Apr 11;28(3):511–22. doi: 10.1007/s11222-017-9744-8 (PMC5807553; doi:10.1007/s11222-017-9744-8)
Supplement: Supplementary file 1 — Supplementary material 1 (pdf 702 KB) [file 11222_2017_9744_MOESM1_ESM.pdf]

Supplement for "Fast Covariance Estimation for Sparse Functional Data"

by

Luo Xiao, Cai Li, William Checkley and Ciprian M. Crainiceanu

Section S.1 discusses the P-spline mean function estimation. Section S.2 provides proofs of Propositions 1 and 2 in the main paper. Section S.3 provides detailed simulation results. Section S.4 gives an application on a child growth data.

S.1 P-spline mean function estimation

S.1.1 Estimation

Given the observed data $\{(y_{ij}, t_{ij}), j = 1, \dots, m_i, i = 1, \dots, n\}$, where t_{ij} is in the unit interval $[0, 1]$, n is the number of subjects, and m_i is the number of observations for subject i, we have $\mathbb{E}(y_{ij}) = f(t_{ij})$. We model the smooth mean function $f(t)$ by basis expansion $\sum_{1\le\kappa\le c} \theta_\kappa B_\kappa(t)$, where $\boldsymbol{\theta} = (\theta_\kappa)_{1\le\kappa\le c}$ is a coefficient vector, $\boldsymbol{B}(t) = \{B_1(t), \dots, B_c(t)\}^T$ is the collection of B-spline basis functions evaluated at sampling point t, and c is the number of interior knots plus the order (degree plus 1) of the B-splines. We use 10 cubic basis functions, i.e., $c = 10$. Stack all the observations as a column vector $\boldsymbol{y} = [\boldsymbol{y}_1^T, \cdots, \boldsymbol{y}_n^T]^T$, where $\boldsymbol{y}_i = (y_1, \cdots, y_{m_i})^T$. Define $\boldsymbol{B}_i$ as the basis matrix $\boldsymbol{B}_i = [\boldsymbol{B}(t_{i1}), \dots, \boldsymbol{B}(t_{im_i})]^T$ and $\boldsymbol{B} = [\boldsymbol{B}_1^T, \dots, \boldsymbol{B}_n^T]^T$. Let $\boldsymbol{D}$ be the second-order differencing matrix, and let λ denote smoothing parameter. The smoother matrix can be constructed by using P-spline (Eilers and Marx, 1996). Moreover, we can add some pre-specified weights for each subject. In this paper, we use $\omega_i = 1/m_i$. Then $\boldsymbol{W}$ is a diagonal matrix with each ω_i repeating m_i times along the diagonal. Therefore, we estimate $\boldsymbol{\theta}$ by minimizing penalized weighted least squares

$$\hat{\boldsymbol{\theta}} = \arg\min_{\boldsymbol{\theta}} (\boldsymbol{y} - \boldsymbol{B}\boldsymbol{\theta})^T \boldsymbol{W} (\boldsymbol{y} - \boldsymbol{B}\boldsymbol{\theta}) + \lambda \|\boldsymbol{D}\boldsymbol{\theta}\|_2^2. \tag{S.1}$$

The fitted mean function is given by $\hat{f}(t) = \boldsymbol{B}(t)^T(\boldsymbol{B}^T\boldsymbol{W}\boldsymbol{B} + \lambda\boldsymbol{D}^T\boldsymbol{D})^{-1}\boldsymbol{B}^T\boldsymbol{W}\boldsymbol{y}$. For simplicity, we write $\tilde{\boldsymbol{S}}(\lambda) = (\boldsymbol{B}^T\boldsymbol{W}\boldsymbol{B} + \lambda\boldsymbol{D}^T\boldsymbol{D})^{-1}\boldsymbol{B}^T\boldsymbol{W}$ and suppress λ. It leads to $\hat{f}(t) = \boldsymbol{B}(t)^T\tilde{\boldsymbol{S}}\boldsymbol{y}$. Next, we have $\mathrm{Var}(\hat{f}(t)) = \boldsymbol{B}(t)^T\tilde{\boldsymbol{S}}\boldsymbol{\Lambda}\tilde{\boldsymbol{S}}^T\boldsymbol{B}(t)$, where $\mathrm{cov}(\boldsymbol{y}) = \boldsymbol{\Lambda}$ is a block diagonal matrix with subject covariance as each block. Specifically, for curve $\boldsymbol{y}_i$, $\mathrm{cov}(\boldsymbol{y}_i) = \boldsymbol{\Lambda}_i + \sigma_\epsilon^2\boldsymbol{I}_{m_i}$ is the covariance of underlying trajectory contaminated by white noise with error variance σ_ϵ^2, where $\boldsymbol{\Lambda}_i$ is the covariance of the ith true trajectory. The confidence band for mean function $f(t)$ can be constructed accordingly. Indeed, a 95% pointwise confidence interval for $f(t)$ is

$$\hat{f}(t) \pm 1.96\sqrt{\boldsymbol{B}(t)^T\tilde{\boldsymbol{S}}\hat{\boldsymbol{\Lambda}}\tilde{\boldsymbol{S}}^T\boldsymbol{B}(t)}.$$

S.1.2 Selection of smoothing parameter

We use leave-one-subject-out cross validation to select smoothing parameter for mean function. A fast algorithm is derived for approximating the leave-one-subject-out cross validation.

Let $\tilde{\boldsymbol{y}}_i^{[i]}$ be the prediction of $\boldsymbol{y}_i$ by applying the proposed method to the data without the data from the ith subject, then the cross-validated error is

$$\text{iCV} = \sum_{i=1}^{n} \|\tilde{\boldsymbol{y}}_i^{[i]} - \boldsymbol{y}_i\|^2. \tag{S.2}$$

Now we introduce a shortcut formula for iCV. First we let $\boldsymbol{S} = \boldsymbol{B}(\boldsymbol{B}^T\boldsymbol{W}\boldsymbol{B} + \lambda\boldsymbol{D}^T\boldsymbol{D})^{-1}\boldsymbol{B}^T\boldsymbol{W}$, which is the smoother matrix for the proposed method. $\boldsymbol{S}$ can be written as $(\boldsymbol{B}\boldsymbol{A})[\boldsymbol{I} + \lambda\text{diag}(\boldsymbol{s})]^{-1}(\boldsymbol{B}\boldsymbol{A})^T\boldsymbol{W}$ for some square matrix $\boldsymbol{A}$ and $\boldsymbol{s}$ is a column vector. Furthermore, both $\boldsymbol{A}$ and $\boldsymbol{s}$ do not depend on λ.

Let $\boldsymbol{S}_i = \boldsymbol{B}_i(\boldsymbol{B}^T\boldsymbol{W}\boldsymbol{B} + \lambda\boldsymbol{D}^T\boldsymbol{D})^{-1}\boldsymbol{B}^T\boldsymbol{W}$ and $\boldsymbol{S}_{ii} = \boldsymbol{B}_i(\boldsymbol{B}^T\boldsymbol{W}\boldsymbol{B} + \lambda\boldsymbol{D}^T\boldsymbol{D})^{-1}\boldsymbol{B}_i^T w_i$. Then $\boldsymbol{S}_i$ is of dimension $m_i \times N$, where $N = \sum_{i=1}^n m_i$, and $\boldsymbol{S}_{ii}$ is symmetric and of dimension $m_i \times m_i$.

Lemma S. 1. *The* iCV *in* (S.2) *can be simplified as*

$$\text{iCV} = \sum_{i=1}^{n} \|(\boldsymbol{I}_{m_i} - \boldsymbol{S}_{ii})^{-1}(\boldsymbol{S}_i\boldsymbol{y} - \boldsymbol{y}_i)\|^2.$$

The proof of Lemma S. 1 is the same as that of Lemma 3.1 in Xu and Huang (2012) and thus is omitted. We further simplify iCV by using the approximation $(\boldsymbol{I}_{m_i} - \boldsymbol{S}_{ii}^T)^{-1}(\boldsymbol{I}_{m_i} - \boldsymbol{S}_{ii})^{-1} = \boldsymbol{I}_{m_i} + \boldsymbol{S}_{ii} + \boldsymbol{S}_{ii}^T$. This approximation leads to the generalized cross validation iGCV,

$$\text{iGCV} = \sum_{i=1}^{n}(\boldsymbol{S}_i\boldsymbol{y} - \boldsymbol{y}_i)^T(\boldsymbol{I}_{m_i} + \boldsymbol{S}_{ii} + \boldsymbol{S}_{ii}^T)(\boldsymbol{S}_i\boldsymbol{y} - \boldsymbol{y}_i) = \|\boldsymbol{y} - \boldsymbol{S}\boldsymbol{y}\|^2 + 2\sum_{i=1}^{n}(\boldsymbol{S}_i\boldsymbol{y} - \boldsymbol{y}_i)^T\boldsymbol{S}_{ii}(\boldsymbol{S}_i\boldsymbol{y} - \boldsymbol{y}_i). \tag{S.3}$$

We further simplify iGCV. Let $\boldsymbol{F}_i = \boldsymbol{B}_i\boldsymbol{A}$, $\boldsymbol{F} = \boldsymbol{B}\boldsymbol{A}$ and $\tilde{\boldsymbol{F}} = \boldsymbol{F}\boldsymbol{W}$. Define $\boldsymbol{f}_i = \boldsymbol{F}_i^T\boldsymbol{y}_i$, $\boldsymbol{f} = \boldsymbol{F}^T\boldsymbol{y}$ and $\tilde{\boldsymbol{f}} = \tilde{\boldsymbol{F}}^T\boldsymbol{y}$. To simplify notation we will denote $[\boldsymbol{I} + \lambda\text{diag}(\boldsymbol{s})]^{-1}$ as $\tilde{\boldsymbol{D}}$, a symmetric matrix, and its diagonal as $\tilde{\boldsymbol{d}}$. Let $\odot$ be the Hadamard product such that for two matrices of the same dimensions $A = (a_{ij})$ and $B = (b_{ij})$, $A \odot B = (a_{ij}b_{ij})$.

Proposition S. 1. *The* iGCV *in* (S.3) *can be simplified as*

$$\text{iGCV} = \|\boldsymbol{y}\|^2 - 2\tilde{\boldsymbol{d}}^T(\tilde{\boldsymbol{f}} \odot \boldsymbol{f}) + (\tilde{\boldsymbol{f}} \odot \tilde{\boldsymbol{d}})^T(\boldsymbol{F}^T\boldsymbol{F})(\tilde{\boldsymbol{f}} \odot \tilde{\boldsymbol{d}}) + 2\tilde{\boldsymbol{d}}^T\boldsymbol{g} - 4\tilde{\boldsymbol{d}}^T\boldsymbol{G}_1\tilde{\boldsymbol{d}} + 2\tilde{\boldsymbol{d}}^T\boldsymbol{G}_2\left\{(\tilde{\boldsymbol{f}} \odot \tilde{\boldsymbol{d}}) \otimes (\tilde{\boldsymbol{f}} \odot \tilde{\boldsymbol{d}})\right\},$$

where $\boldsymbol{g} = \sum_{i=1}^n w_i\boldsymbol{f}_i \odot \boldsymbol{f}_i$, $\boldsymbol{G}_1 = \sum_{i=1}^n w_i(\boldsymbol{f}_i\tilde{\boldsymbol{f}}^T) \odot (\boldsymbol{F}_i^T\boldsymbol{F}_i)$, *and* $\boldsymbol{G}_2 = \sum_{i=1}^n w_i(\boldsymbol{F}_i^T\boldsymbol{F}_i) \circ (\boldsymbol{F}_i^T\boldsymbol{F}_i)$. *Here* $\circ$ *is the row-wise Khatri-Rao product such that for two matrices with the same number of rows* $A = [\boldsymbol{a}_1, \ldots, \boldsymbol{a}_m]^T$ *and* $B = [\boldsymbol{b}_1, \ldots, \boldsymbol{b}_m]^T$, $A \circ B = [\boldsymbol{a}_1 \otimes \boldsymbol{b}_1, \ldots, \boldsymbol{a}_m \otimes \boldsymbol{b}_m]^T$.

Remark S. 1. *While the above formula looks complex, it can be efficiently computed. Indeed, only the term* $\tilde{\boldsymbol{d}}$ *depend on the smoothing parameter* λ *and it can be easily computed; all other terms including* $\boldsymbol{g}$, $\boldsymbol{G}_1$, $\boldsymbol{G}_2$ *can be pre-calculated just for once.*

S.1.3 Proof of Proposition S.1

Proof. We will use the following Lemma (page 241, Seber 2007).

Lemma S. 2. *Let* $\boldsymbol{A}$, $\boldsymbol{B}$ *and* $\boldsymbol{C}$ *and* $\boldsymbol{D}$ *be compatible matrices. Then*

$$\mathrm{tr}(\boldsymbol{ABCD}) = (\mathrm{vec}\,\boldsymbol{D})^T(\boldsymbol{A}\otimes\boldsymbol{C}^T)\mathrm{vec}\,\boldsymbol{B}^T.$$

We write iGCV as a sum

$$\mathrm{iGCV} = \mathcal{I} + 2\sum_{i=1}^{n}(\mathcal{II}_i + 2\mathcal{III}_i + \mathcal{IV}_i), \tag{S.4}$$

where $\mathcal{I} = \|\boldsymbol{y}-\boldsymbol{Sy}\|^2$, $\mathcal{II}_i = \boldsymbol{y}_i^T\boldsymbol{S}_{ii}\boldsymbol{y}_i$, $\mathcal{III}_i = (\boldsymbol{S}_i\boldsymbol{y})^T\boldsymbol{S}_{ii}\boldsymbol{y}_i$ and $\mathcal{IV}_i = (\boldsymbol{S}_i\boldsymbol{y})^T\boldsymbol{S}_{ii}(\boldsymbol{S}_i\boldsymbol{y})$. Note that we have the following equalities that will be used later:

$$\boldsymbol{S} = (\boldsymbol{BA})[\boldsymbol{I}+\lambda\mathrm{diag}(\boldsymbol{s})]^{-1}(\boldsymbol{BA})^T\boldsymbol{W} = \boldsymbol{F}\tilde{\boldsymbol{D}}\tilde{\boldsymbol{F}}^T,$$

$$\boldsymbol{S}_i = (\boldsymbol{B}_i\boldsymbol{A})[\boldsymbol{I}+\lambda\mathrm{diag}(\boldsymbol{s})]^{-1}(\boldsymbol{BA})^T\boldsymbol{W} = \boldsymbol{F}_i\tilde{\boldsymbol{D}}\tilde{\boldsymbol{F}}^T,$$

$$\boldsymbol{S}_{ii} = (\boldsymbol{B}_i\boldsymbol{A})[\boldsymbol{I}+\lambda\mathrm{diag}(\boldsymbol{s})]^{-1}(\boldsymbol{B}_i\boldsymbol{A})^T w_i = w_i\boldsymbol{F}_i\tilde{\boldsymbol{D}}\boldsymbol{F}_i^T.$$

We first compute $\mathcal{I}$. We have

$$\mathcal{I} = \|\boldsymbol{y}-\boldsymbol{Sy}\|^2 = \|\boldsymbol{y}-\boldsymbol{F}\tilde{\boldsymbol{D}}\tilde{\boldsymbol{f}}\|^2 = \|\boldsymbol{y}\|^2 - 2\boldsymbol{f}^T\tilde{\boldsymbol{D}}\tilde{\boldsymbol{f}} + \tilde{\boldsymbol{f}}^T\tilde{\boldsymbol{D}}\boldsymbol{F}^T\boldsymbol{F}\tilde{\boldsymbol{D}}\tilde{\boldsymbol{f}}.$$

Thus,

$$\mathcal{I} = \|\boldsymbol{y}\|^2 - 2\tilde{\boldsymbol{d}}^T(\tilde{\boldsymbol{f}}\odot\boldsymbol{f}) + (\tilde{\boldsymbol{f}}\odot\tilde{\boldsymbol{d}})^T(\boldsymbol{F}^T\boldsymbol{F})(\tilde{\boldsymbol{f}}\odot\tilde{\boldsymbol{d}}). \tag{S.5}$$

Second, we compute $\mathcal{II}_i$. We have

$$\mathcal{II}_i = \boldsymbol{y}_i^T\boldsymbol{S}_{ii}\boldsymbol{y}_i = w_i\boldsymbol{f}_i^T\tilde{\boldsymbol{D}}\boldsymbol{f}_i = w_i\tilde{\boldsymbol{d}}^T(\boldsymbol{f}_i\odot\boldsymbol{f}_i). \tag{S.6}$$

Third, we compute $\mathcal{III}_i$. Note that $\boldsymbol{S}_i\boldsymbol{y} = \boldsymbol{F}_i\tilde{\boldsymbol{D}}\tilde{\boldsymbol{f}}$ and hence

$$\mathcal{III}_i = (\boldsymbol{S}_i\boldsymbol{y})^T\boldsymbol{S}_{ii}\boldsymbol{y}_i = w_i\tilde{\boldsymbol{f}}^T\tilde{\boldsymbol{D}}\boldsymbol{F}_i^T\boldsymbol{F}_i\tilde{\boldsymbol{D}}\boldsymbol{f}_i = w_i\mathrm{tr}(\boldsymbol{f}_i\tilde{\boldsymbol{f}}^T\tilde{\boldsymbol{D}}\boldsymbol{F}_i^T\boldsymbol{F}_i\tilde{\boldsymbol{D}}) = w_i\tilde{\boldsymbol{d}}^T\left\{(\boldsymbol{f}_i\tilde{\boldsymbol{f}}^T)\odot(\boldsymbol{F}_i^T\boldsymbol{F}_i)\right\}\tilde{\boldsymbol{d}}.$$

Thus we have

$$\mathcal{III}_i = w_i\tilde{\boldsymbol{d}}^T\left\{(\boldsymbol{f}_i\tilde{\boldsymbol{f}}^T)\odot(\boldsymbol{F}_i^T\boldsymbol{F}_i)\right\}\tilde{\boldsymbol{d}}. \tag{S.7}$$

Fourth, we compute $\mathcal{IV}_i$. We derive that

$$\begin{aligned}
\mathcal{IV}_i &= (\boldsymbol{S}_i\boldsymbol{y})^T\boldsymbol{S}_{ii}(\boldsymbol{S}_i\boldsymbol{y}) \\
&= w_i\tilde{\boldsymbol{f}}^T\tilde{\boldsymbol{D}}\boldsymbol{F}_i^T\boldsymbol{F}_i\tilde{\boldsymbol{D}}\boldsymbol{F}_i^T\boldsymbol{F}_i\tilde{\boldsymbol{D}}\tilde{\boldsymbol{f}} \\
&= w_i\mathrm{tr}(\tilde{\boldsymbol{f}}^T\tilde{\boldsymbol{D}}\boldsymbol{F}_i^T\boldsymbol{F}_i\tilde{\boldsymbol{D}}\boldsymbol{F}_i^T\boldsymbol{F}_i\tilde{\boldsymbol{D}}\tilde{\boldsymbol{f}}) \\
&= w_i\mathrm{tr}(\boldsymbol{F}_i^T\boldsymbol{F}_i\tilde{\boldsymbol{D}}\boldsymbol{F}_i^T\boldsymbol{F}_i\tilde{\boldsymbol{D}}\tilde{\boldsymbol{f}}\tilde{\boldsymbol{f}}^T\tilde{\boldsymbol{D}}) \\
&= w_i\mathrm{tr}\left\{\tilde{\boldsymbol{D}}(\boldsymbol{F}_i^T\boldsymbol{F}_i)(\tilde{\boldsymbol{f}}\odot\tilde{\boldsymbol{d}})(\tilde{\boldsymbol{f}}\odot\tilde{\boldsymbol{d}})^T(\boldsymbol{F}_i^T\boldsymbol{F}_i)\right\} \\
&= w_i\tilde{\boldsymbol{d}}^T\left[\left\{(\boldsymbol{F}_i^T\boldsymbol{F}_i)(\tilde{\boldsymbol{f}}\odot\tilde{\boldsymbol{d}})\right\}\odot\left\{(\boldsymbol{F}_i^T\boldsymbol{F}_i)(\tilde{\boldsymbol{f}}\odot\tilde{\boldsymbol{d}})\right\}\right].
\end{aligned}$$

Hence we obtain

$$\mathcal{IV}_i = w_i\tilde{\boldsymbol{d}}^T\left\{(\boldsymbol{F}_i^T\boldsymbol{F}_i)\circ(\boldsymbol{F}_i^T\boldsymbol{F}_i)\right\}\left\{(\tilde{\boldsymbol{f}}\odot\tilde{\boldsymbol{d}})\otimes(\tilde{\boldsymbol{f}}\odot\tilde{\boldsymbol{d}})\right\}. \tag{S.8}$$

Now with (S.4), (S.5), (S.6), (S.7) and (S.8), we obtain that

$$\begin{aligned}
\mathrm{iGCV} &= \|\boldsymbol{y}\|^2 - 2\tilde{\boldsymbol{d}}^T(\tilde{\boldsymbol{f}}\odot\boldsymbol{f}) + (\tilde{\boldsymbol{f}}\odot\tilde{\boldsymbol{d}})^T(\boldsymbol{F}^T\boldsymbol{F})(\tilde{\boldsymbol{f}}\odot\tilde{\boldsymbol{d}}) + 2\sum_{i=1}^n w_i\tilde{\boldsymbol{d}}^T(\boldsymbol{f}_i\odot\boldsymbol{f}_i) \\
&+ 2\sum_{i=1}^n\left[2w_i\tilde{\boldsymbol{d}}^T\left\{(\boldsymbol{f}_i\tilde{\boldsymbol{f}}^T)\odot(\boldsymbol{F}_i^T\boldsymbol{F}_i)\right\}\tilde{\boldsymbol{d}} + w_i\tilde{\boldsymbol{d}}^T\left\{(\boldsymbol{F}_i^T\boldsymbol{F}_i)\circ(\boldsymbol{F}_i^T\boldsymbol{F}_i)\right\}\left\{(\tilde{\boldsymbol{f}}\odot\tilde{\boldsymbol{d}})\otimes(\tilde{\boldsymbol{f}}\odot\tilde{\boldsymbol{d}})\right\}\right] \\
&= \|\boldsymbol{y}\|^2 - 2\tilde{\boldsymbol{d}}^T(\tilde{\boldsymbol{f}}\odot\boldsymbol{f}) + (\tilde{\boldsymbol{f}}\odot\tilde{\boldsymbol{d}})^T(\boldsymbol{F}^T\boldsymbol{F})(\tilde{\boldsymbol{f}}\odot\tilde{\boldsymbol{d}}) + 2\tilde{\boldsymbol{d}}^T\left\{\sum_{i=1}^n w_i\boldsymbol{f}_i\odot\boldsymbol{f}_i\right\} \\
&- 4\tilde{\boldsymbol{d}}^T\left\{\sum_{i=1}^n w_i(\boldsymbol{f}_i\tilde{\boldsymbol{f}}^T)\odot(\boldsymbol{F}_i^T\boldsymbol{F}_i)\right\}\tilde{\boldsymbol{d}} + 2\tilde{\boldsymbol{d}}^T\left\{\sum_{i=1}^n w_i(\boldsymbol{F}_i^T\boldsymbol{F}_i)\circ(\boldsymbol{F}_i^T\boldsymbol{F}_i)\right\}\left\{(\tilde{\boldsymbol{f}}\odot\tilde{\boldsymbol{d}})\otimes(\tilde{\boldsymbol{f}}\odot\tilde{\boldsymbol{d}})\right\},
\end{aligned}$$

which completes the proof. □

S.2 Proofs of Proposition 1 and Proposition 2

Proof of Proposition 1: We will use the following Lemma (Isserlis, 1918) in our proof.

Lemma S. 3. *If $(x_1,\cdots,x_{2n})$ is a zero mean multivariate normal random vector, then*

$$\mathbb{E}(x_1,\cdots,x_{2n}) = \sum\prod\mathbb{E}(x_ix_j),$$

where x_ix_j are all distinct pairs over $x_1,x_2,\cdots,x_{2n}$.

First, we have

$$\begin{aligned}
\mathrm{cov}(C_{ijj'},C_{ikk'}) &= \mathbb{E}(C_{ijj'}C_{ikk'}) - \mathbb{E}(C_{ijj'})\mathbb{E}(C_{ikk'}), \\
\mathbb{E}(C_{ijj'}) &= \mathcal{C}(t_{ij},t_{ij'}) + \delta_{jj'}\sigma_\epsilon^2, \\
\mathbb{E}(C_{ikk'}) &= \mathcal{C}(t_{ik},t_{ik'}) + \delta_{kk'}\sigma_\epsilon^2.
\end{aligned}$$

Then,

$$\mathbb{E}(C_{ijj'})\mathbb{E}(C_{ikk'}) = \mathcal{C}(t_{ij}, t_{ij'})\mathcal{C}(t_{ik}, t_{ik'}) + \mathcal{C}(t_{ij}, t_{ij'})\delta_{kk'}\sigma_\epsilon^2 + \mathcal{C}(t_{ik}, t_{ik'})\delta_{jj'}\sigma_\epsilon^2 + \delta_{jj'}\sigma_\epsilon^2\delta_{kk'}\sigma_\epsilon^2.$$

Next, we derive $\mathbb{E}(C_{ijj'}C_{ikk'})$ that

$$\begin{aligned}
\mathbb{E}(C_{ijj'}C_{ikk'}) &= \mathbb{E}(y_{ij}y_{ij'}y_{ik}y_{ik'}) \\
&= \mathbb{E}\{(x_i(t_{ij}) + \epsilon_{ij})(x_i(t_{ij'}) + \epsilon_{ij'})(x_i(t_{ik}) + \epsilon_{ik})(x_i(t_{ik'}) + \epsilon_{ik'})\} \\
&= \mathbb{E}\{x_i(t_{ij})x_i(t_{ij'})x_i(t_{ik})x_i(t_{ik'})\} + \mathbb{E}\{x_i(t_{ij})x_i(t_{ij'})x_i(t_{ik})\}\mathbb{E}(\epsilon_{ik'}) \\
&+ \mathbb{E}\{x_i(t_{ij})x_i(t_{ij'})x_i(t_{ik'})\}\mathbb{E}(\epsilon_{ik}) + \mathbb{E}\{x_i(t_{ij})x_i(t_{ij'})\}\mathbb{E}(\epsilon_{ik}\epsilon_{ik'}) \\
&+ \mathbb{E}\{x_i(t_{ij})x_i(t_{ik})x_i(t_{ik'})\}\mathbb{E}(\epsilon_{ij'}) + \mathbb{E}\{x_i(t_{ij})x_i(t_{ik})\}\mathbb{E}(\epsilon_{ij'}\epsilon_{ik'}) \\
&+ \mathbb{E}\{x_i(t_{ij})x_i(t_{ik'})\}\mathbb{E}(\epsilon_{ij'}\epsilon_{ik}) + \mathbb{E}\{x_i(t_{ij})\}\mathbb{E}(\epsilon_{ij'}\epsilon_{ik}\epsilon_{ik'}) \\
&+ \mathbb{E}\{x_i(t_{ij'})x_i(t_{ik})x_i(t_{ik'})\}\mathbb{E}(\epsilon_{ij}) + \mathbb{E}\{x_i(t_{ij'})x_i(t_{ik})\}\mathbb{E}(\epsilon_{ij}\epsilon_{ik'}) \\
&+ \mathbb{E}\{x_i(t_{ij'})x_i(t_{ik'})\}\mathbb{E}(\epsilon_{ij}\epsilon_{ik}) + \mathbb{E}\{x_i(t_{ij'})\}\mathbb{E}(\epsilon_{ij}\epsilon_{ik}\epsilon_{ik'}) \\
&+ \mathbb{E}\{x_i(t_{ik})x_i(t_{ik'})\}\mathbb{E}(\epsilon_{ij}\epsilon_{ij'}) + \mathbb{E}\{x_i(t_{ik})\}\mathbb{E}(\epsilon_{ij}\epsilon_{ij'}\epsilon_{ik'}) \\
&+ \mathbb{E}\{x_i(t_{ik'})\}\mathbb{E}(\epsilon_{ij}\epsilon_{ij'}\epsilon_{ik}) + \mathbb{E}(\epsilon_{ij}\epsilon_{ij'}\epsilon_{ik}\epsilon_{ik'}) \\
&= \mathbb{E}\{x_i(t_{ij})x_i(t_{ij'})x_i(t_{ik})x_i(t_{ik'})\} + \mathbb{E}(\epsilon_{ij}\epsilon_{ij'}\epsilon_{ik}\epsilon_{ik'}) \\
&+ \mathcal{C}(t_{ij}, t_{ij'})\delta_{kk'}\sigma_\epsilon^2 + \mathcal{C}(t_{ij}, t_{ik})\delta_{j'k'}\sigma_\epsilon^2 + \mathcal{C}(t_{ij}, t_{ik'})\delta_{j'k}\sigma_\epsilon^2 \\
&+ \mathcal{C}(t_{ij'}, t_{ik})\delta_{jk'}\sigma_\epsilon^2 + \mathcal{C}(t_{ij'}, t_{ik'})\delta_{jk}\sigma_\epsilon^2 + \mathcal{C}(t_{ik}, t_{ik'})\delta_{jj'}\sigma_\epsilon^2.
\end{aligned}$$

Finally, with $\mathbb{E}(C_{ijj'})\mathbb{E}(C_{ikk'})$ and $\mathbb{E}(C_{ijj'}C_{ikk'})$, we have

$$\begin{aligned}
\operatorname{cov}(C_{ijj'}, C_{ikk'}) &= \mathcal{C}(t_{ij}, t_{ij'})\mathcal{C}(t_{ik}, t_{ik'}) + \mathcal{C}(t_{ij}, t_{ik})\mathcal{C}(t_{ij'}, t_{ik'}) + \mathcal{C}(t_{ij}, t_{ik'})\mathcal{C}(t_{ij'}, t_{ik}) \\
&+ \delta_{jj'}\sigma_\epsilon^2\delta_{kk'}\sigma_\epsilon^2 + \delta_{jk}\sigma_\epsilon^2\delta_{j'k'}\sigma_\epsilon^2 + \delta_{jk'}\sigma_\epsilon^2\delta_{j'k}\sigma_\epsilon^2 \\
&+ \mathcal{C}(t_{ij}, t_{ij'})\delta_{kk'}\sigma_\epsilon^2 + \mathcal{C}(t_{ij}, t_{ik})\delta_{j'k'}\sigma_\epsilon^2 + \mathcal{C}(t_{ij}, t_{ik'})\delta_{j'k}\sigma_\epsilon^2 \\
&+ \mathcal{C}(t_{ij'}, t_{ik})\delta_{jk'}\sigma_\epsilon^2 + \mathcal{C}(t_{ij'}, t_{ik'})\delta_{jk}\sigma_\epsilon^2 + \mathcal{C}(t_{ik}, t_{ik'})\delta_{jj'}\sigma_\epsilon^2 \\
&- \mathcal{C}(t_{ij}, t_{ij'})\mathcal{C}(t_{ik}, t_{ik'}) - \mathcal{C}(t_{ij}, t_{ij'})\delta_{kk'}\sigma_\epsilon^2 - \mathcal{C}(t_{ik}, t_{ik'})\delta_{jj'}\sigma_\epsilon^2 - \delta_{jj'}\sigma_\epsilon^2\delta_{kk'}\sigma_\epsilon^2 \\
&= \mathcal{C}(t_{ij}, t_{ik})\mathcal{C}(t_{ij'}, t_{ik'}) + \mathcal{C}(t_{ij}, t_{ik'})\mathcal{C}(t_{ij'}, t_{ik}) + \delta_{jk}\delta_{j'k'}\sigma_\epsilon^4 + \delta_{jk'}\delta_{j'k}\sigma_\epsilon^4 \\
&+ \mathcal{C}(t_{ij}, t_{ik})\delta_{j'k'}\sigma_\epsilon^2 + \mathcal{C}(t_{ij}, t_{ik'})\delta_{j'k}\sigma_\epsilon^2 + \mathcal{C}(t_{ij'}, t_{ik})\delta_{jk'}\sigma_\epsilon^2 + \mathcal{C}(t_{ij'}, t_{ik'})\delta_{jk}\sigma_\epsilon^2.
\end{aligned}$$

By the definition $\boldsymbol{M}_{ijk} = \left\{\mathcal{C}(t_{ij}, t_{ijk}), \delta_{jk}\sigma_\epsilon^2\right\}^T$, we derive that

$$\begin{aligned}
\operatorname{cov}(C_{ijj'}, C_{ikk'}) &= \left\{\mathcal{C}(t_{ij}, t_{ik})\mathcal{C}(t_{ij'}, t_{ik'}) + \mathcal{C}(t_{ij}, t_{ik})\delta_{j'k'}\sigma_\epsilon^2 + \mathcal{C}(t_{ij'}, t_{ik'})\delta_{jk}\sigma_\epsilon^2 + \delta_{jk}\delta_{j'k'}\sigma_\epsilon^4\right\} \\
&+ \left\{\mathcal{C}(t_{ij}, t_{ik'})\mathcal{C}(t_{ij'}, t_{ik}) + \mathcal{C}(t_{ij}, t_{ik'})\delta_{j'k}\sigma_\epsilon^2 + \mathcal{C}(t_{ij'}, t_{ik})\delta_{jk'}\sigma_\epsilon^2 + \delta_{jk'}\delta_{j'k}\sigma_\epsilon^4\right\} \\
&= \mathbf{1}^T(\boldsymbol{M}_{ij_1j_3} \otimes \boldsymbol{M}_{ij_2j_4} + \boldsymbol{M}_{ij_1j_4} \otimes \boldsymbol{M}_{ij_2j_3}),
\end{aligned}$$

which proves the proposition.

Proof of Proposition 2: We first write iGCV as a sum

$$\text{iGCV} = \mathcal{I} + 2\sum_{i=1}^{n}(\mathcal{II}_i - 2\mathcal{III}_i + \mathcal{IV}_i), \tag{S.9}$$

where $\mathcal{I} = \|\hat{\boldsymbol{C}} - \boldsymbol{S}\hat{\boldsymbol{C}}\|^2$, $\mathcal{II}_i = \hat{\boldsymbol{C}}_i^T \boldsymbol{S}_{ii}\hat{\boldsymbol{C}}_i$, $\mathcal{III}_i = (\boldsymbol{S}_i\hat{\boldsymbol{C}})^T \boldsymbol{S}_{ii}\hat{\boldsymbol{C}}_i$ and $\mathcal{IV}_i = (\boldsymbol{S}_i\hat{\boldsymbol{C}})^T \boldsymbol{S}_{ii}(\boldsymbol{S}_i\hat{\boldsymbol{C}})$.

Note that we have the following equalities that will be used later:

$$\boldsymbol{S} = (\boldsymbol{X}\boldsymbol{A})[\boldsymbol{I} + \lambda \text{diag}(\boldsymbol{s})]^{-1}(\boldsymbol{X}\boldsymbol{A})^T\boldsymbol{W} = \boldsymbol{F}\tilde{\boldsymbol{D}}\tilde{\boldsymbol{F}},$$

$$\boldsymbol{S}_i = (\boldsymbol{X}_i\boldsymbol{A})[\boldsymbol{I} + \lambda \text{diag}(\boldsymbol{s})]^{-1}(\boldsymbol{X}\boldsymbol{A})^T\boldsymbol{W} = \boldsymbol{F}_i\tilde{\boldsymbol{D}}\tilde{\boldsymbol{F}},$$

$$\boldsymbol{S}_{ii} = (\boldsymbol{X}_i\boldsymbol{A})[\boldsymbol{I} + \lambda \text{diag}(\boldsymbol{s})]^{-1}(\boldsymbol{X}_i\boldsymbol{A})^T\boldsymbol{W}_i = \boldsymbol{F}_i\tilde{\boldsymbol{D}}\boldsymbol{F}_i^T\boldsymbol{W}_i.$$

We first compute $\mathcal{I}$. We have

$$\mathcal{I} = \|\hat{\boldsymbol{C}} - \boldsymbol{S}\hat{\boldsymbol{C}}\|^2 = \|\hat{\boldsymbol{C}} - \boldsymbol{F}\tilde{\boldsymbol{D}}\tilde{\boldsymbol{f}}\|^2 = \|\hat{\boldsymbol{C}}\|^2 - 2\boldsymbol{f}^T\tilde{\boldsymbol{D}}\tilde{\boldsymbol{f}} + \tilde{\boldsymbol{f}}^T\tilde{\boldsymbol{D}}\boldsymbol{F}^T\boldsymbol{F}\tilde{\boldsymbol{D}}\tilde{\boldsymbol{f}}.$$

Thus,

$$\mathcal{I} = \|\hat{\boldsymbol{C}}\|^2 - 2\tilde{\boldsymbol{d}}^T(\tilde{\boldsymbol{f}} \odot \boldsymbol{f}) + (\tilde{\boldsymbol{f}} \odot \tilde{\boldsymbol{d}})^T(\boldsymbol{F}^T\boldsymbol{F})(\tilde{\boldsymbol{f}} \odot \tilde{\boldsymbol{d}}). \tag{S.10}$$

Second, we compute $\mathcal{II}_i$. We have

$$\mathcal{II}_i = \hat{\boldsymbol{C}}_i^T\boldsymbol{S}_{ii}\hat{\boldsymbol{C}}_i = \boldsymbol{f}_i^T\tilde{\boldsymbol{D}}\boldsymbol{F}_i^T\boldsymbol{W}_i\hat{\boldsymbol{C}}_i = \boldsymbol{f}_i^T\tilde{\boldsymbol{D}}\boldsymbol{J}_i = \tilde{\boldsymbol{d}}^T(\boldsymbol{J}_i \odot \boldsymbol{f}_i). \tag{S.11}$$

Third, we compute $\mathcal{III}_i$. Note that $\boldsymbol{S}_i\hat{\boldsymbol{C}} = \boldsymbol{F}_i\tilde{\boldsymbol{D}}\tilde{\boldsymbol{f}}$ and hence

$$\mathcal{III}_i = (\boldsymbol{S}_i\hat{\boldsymbol{C}})^T\boldsymbol{S}_{ii}\hat{\boldsymbol{C}}_i = \tilde{\boldsymbol{f}}^T\tilde{\boldsymbol{D}}\boldsymbol{F}_i^T\boldsymbol{F}_i\tilde{\boldsymbol{D}}\boldsymbol{J}_i = \text{tr}(\boldsymbol{J}_i\tilde{\boldsymbol{f}}^T\tilde{\boldsymbol{D}}\boldsymbol{F}_i^T\boldsymbol{F}_i\tilde{\boldsymbol{D}}) = \tilde{\boldsymbol{d}}^T\{(\boldsymbol{J}_i\tilde{\boldsymbol{f}}^T) \odot (\boldsymbol{F}_i^T\boldsymbol{F}_i)\}\tilde{\boldsymbol{d}}.$$

Thus we have

$$\mathcal{III}_i = \tilde{\boldsymbol{d}}^T\{(\boldsymbol{J}_i\tilde{\boldsymbol{f}}^T) \odot (\boldsymbol{F}_i^T\boldsymbol{F}_i)\}\tilde{\boldsymbol{d}}. \tag{S.12}$$

Fourth, we compute $\mathcal{IV}_i$. We derive that

$$\begin{aligned}
\mathcal{IV}_i &= (\boldsymbol{S}_i\hat{\boldsymbol{C}})^T\boldsymbol{S}_{ii}(\boldsymbol{S}_i\hat{\boldsymbol{C}}) \\
&= \tilde{\boldsymbol{f}}^T\tilde{\boldsymbol{D}}\boldsymbol{F}_i^T\boldsymbol{F}_i\tilde{\boldsymbol{D}}\boldsymbol{F}_i^T\boldsymbol{W}_i\boldsymbol{F}_i\tilde{\boldsymbol{D}}\tilde{\boldsymbol{f}} \\
&= (\tilde{\boldsymbol{f}} \odot \tilde{\boldsymbol{d}})^T(\boldsymbol{F}_i^T\boldsymbol{F}_i)\tilde{\boldsymbol{D}}(\boldsymbol{F}_i^T\boldsymbol{W}_i\boldsymbol{F}_i)(\tilde{\boldsymbol{f}} \odot \tilde{\boldsymbol{d}}).
\end{aligned}$$

Hence we obtain

$$\mathcal{IV}_i = \tilde{\boldsymbol{d}}^T\left[\left\{(\boldsymbol{F}_i^T\boldsymbol{F}_i)(\tilde{\boldsymbol{f}} \odot \tilde{\boldsymbol{d}})\right\} \odot \left\{(\boldsymbol{F}_i^T\boldsymbol{W}_i\boldsymbol{F}_i)(\tilde{\boldsymbol{f}} \odot \tilde{\boldsymbol{d}})\right\}\right]. \tag{S.13}$$

Now with (S.9), (S.10), (S.11), (S.12) and (S.13), we obtain that

$$\begin{aligned}
\text{iGCV} &= \|\hat{\boldsymbol{C}}\|^2 - 2\tilde{\boldsymbol{d}}^T(\tilde{\boldsymbol{f}} \odot \boldsymbol{f}) + (\tilde{\boldsymbol{f}} \odot \tilde{\boldsymbol{d}})^T(\boldsymbol{F}^T\boldsymbol{F})(\tilde{\boldsymbol{f}} \odot \tilde{\boldsymbol{d}}) + 2\sum_{i=1}^{n} \tilde{\boldsymbol{d}}^T(\boldsymbol{J}_i \odot \boldsymbol{f}_i) \\
&\quad + 2\sum_{i=1}^{n}\left[-2\tilde{\boldsymbol{d}}^T\left\{(\boldsymbol{J}_i\tilde{\boldsymbol{f}}^T) \circ (\boldsymbol{F}_i^T\boldsymbol{F}_i)\right\}\tilde{\boldsymbol{d}} + \tilde{\boldsymbol{d}}^T\left\{\boldsymbol{L}_i \odot (\boldsymbol{F}_i^T\boldsymbol{F}_i)\right\}\left\{(\tilde{\boldsymbol{f}} \odot \tilde{\boldsymbol{d}}) \otimes (\tilde{\boldsymbol{f}} \odot \tilde{\boldsymbol{d}})\right\}\right] \\
&= \|\hat{\boldsymbol{C}}\|^2 - 2\tilde{\boldsymbol{d}}^T(\tilde{\boldsymbol{f}} \odot \boldsymbol{f}) + (\tilde{\boldsymbol{f}} \odot \tilde{\boldsymbol{d}})^T(\boldsymbol{F}^T\boldsymbol{F})(\tilde{\boldsymbol{f}} \odot \tilde{\boldsymbol{d}}) + 2\tilde{\boldsymbol{d}}^T\left\{\sum_{i=1}^{n}\boldsymbol{J}_i \odot \boldsymbol{f}_i\right\} \\
&\quad - 4\tilde{\boldsymbol{d}}^T\left\{\sum_{i=1}^{n}(\boldsymbol{J}_i\tilde{\boldsymbol{f}}^T) \circ (\boldsymbol{F}_i^T\boldsymbol{F}_i)\right\}\tilde{\boldsymbol{d}} + 2\tilde{\boldsymbol{d}}^T\left[\sum_{i=1}^{n}\left\{(\boldsymbol{F}_i^T\boldsymbol{F}_i)(\tilde{\boldsymbol{f}} \odot \tilde{\boldsymbol{d}})\right\} \odot \left\{(\boldsymbol{F}_i^T\boldsymbol{W}_i\boldsymbol{F}_i)(\tilde{\boldsymbol{f}} \odot \tilde{\boldsymbol{d}})\right\}\right].
\end{aligned}$$

which proves the proposition.

S.3 Additional simulation results

In this subsection, we provide additional simulation results. The summaries in terms of median and standard deviation of ISEs for estimating covariance functions are provided in Table S.1. The summaries in terms of median and IQR of ISEs for estimating the 1st eigenfunction are provided in Table S.2. The summaries in terms of median and IQR of ISEs for estimating the 2nd eigenfunction are provided in Table S.3. The summaries in terms of median and IQR of ISEs for estimating the 3rd eigenfunction are provided in Table S.4. The summaries in terms of median and IQR of SEs for estimating the 1st eigenvalue are provided in Table S.5. The summaries in terms of median and IQR of SEs for estimating the 2nd eigenvalue are provided in Table S.6. The summaries in terms of median and IQR of SEs for estimating the 3rd eigenvalue are provided in Table S.7. The summaries in terms of median and IQR of computation times for estimating covariance functions are provided in Table S.8.

For the three additional competitors, 1) FACEs(1-Stage) only estimates the covariance function with independence assumption, that is, we don't take account of correlation structure in the estimation procedure but keep other default settings unchanged; 2) TPRS(50) uses $m + 50$ as the number of knots, where m is the dimension of the null space; 3) TPRS(97) uses $m + 97$ as the number of knots.

S.4 Additional application: child growth data

The Contents study was conducted in Pampas de San Juan Miraflores and Nuevo Paraso, two peri-urban shanty towns with high population density, 25 km south of central Lima. These peri-urban communities are comprised of 50,000 residents, the majority of whom are immigrants from rural areas of the Peruvian Andes who settled nearly 35 years ago and later claimed unused land on the outskirts of Lima. In the

Table S.1: Median and IQR (in parenthesis) of ISEs of eight estimators for estimating the covariance functions. The results are based on 200 replications.

Case	n	m	SNR	FACEs	FACEs(1-Stage)	TPRS	TPRS(50)	TPRS(97)	*fpca.sc*	MLE	*loc*
Case 1	100	5	2	**0.169** (0.085)	0.257 (0.118)	0.460 (0.129)	0.309 (0.152)	0.332 (0.168)	0.305 (0.170)	0.244 (0.224)	0.302 (0.226)
	400	5	2	**0.060** (0.028)	0.083 (0.036)	0.319 (0.041)	0.113 (0.051)	0.132 (0.061)	0.122 (0.053)	0.102 (0.077)	0.307 (0.071)
	100	10	2	**0.094** (0.050)	0.150 (0.076)	0.363 (0.080)	0.165 (0.086)	0.197 (0.097)	0.184 (0.089)	0.143 (0.129)	0.221 (0.093)
	400	10	2	**0.034** (0.019)	0.045 (0.024)	0.285 (0.022)	0.051 (0.023)	0.061 (0.025)	0.057 (0.023)	0.069 (0.050)	0.226 (0.062)
	100	5	5	**0.116** (0.070)	0.207 (0.116)	0.419 (0.085)	0.262 (0.113)	0.280 (0.132)	0.255 (0.116)	0.144 (0.125)	0.302 (0.230)
	400	5	5	**0.034** (0.017)	0.065 (0.026)	0.305 (0.029)	0.086 (0.036)	0.103 (0.038)	0.094 (0.038)	0.079 (0.060)	0.313 (0.067)
	100	10	5	**0.068** (0.056)	0.121 (0.064)	0.346 (0.066)	0.141 (0.075)	0.166 (0.081)	0.154 (0.075)	0.125 (0.094)	0.203 (0.096)
	400	10	5	**0.018** (0.011)	0.036 (0.021)	0.280 (0.017)	0.044 (0.019)	0.050 (0.021)	0.047 (0.021)	0.063 (0.042)	0.229 (0.062)
Case 2	100	5	2	**0.047** (0.017)	0.059 (0.023)	0.061 (0.024)	0.069 (0.035)	0.072 (0.042)	0.070 (0.040)	0.090 (0.053)	0.049 (0.015)
	400	5	2	**0.019** (0.006)	0.024 (0.008)	0.031 (0.009)	0.030 (0.012)	0.032 (0.015)	0.030 (0.015)	0.028 (0.010)	0.029 (0.006)
	100	10	2	**0.025** (0.010)	0.034 (0.012)	0.041 (0.013)	0.042 (0.017)	0.049 (0.022)	0.047 (0.022)	0.040 (0.017)	0.034 (0.009)
	400	10	2	**0.009** (0.003)	0.013 (0.003)	0.022 (0.003)	0.017 (0.004)	0.017 (0.005)	0.016 (0.005)	0.015 (0.004)	0.021 (0.004)
	100	5	5	**0.038** (0.017)	0.049 (0.023)	0.054 (0.020)	0.056 (0.023)	0.062 (0.029)	0.060 (0.030)	0.066 (0.038)	0.043 (0.014)
	400	5	5	**0.014** (0.004)	0.019 (0.005)	0.027 (0.006)	0.025 (0.007)	0.025 (0.008)	0.024 (0.009)	0.023 (0.008)	0.027 (0.004)
	100	10	5	**0.020** (0.006)	0.030 (0.009)	0.035 (0.010)	0.035 (0.013)	0.039 (0.015)	0.038 (0.016)	0.033 (0.012)	0.032 (0.009)
	400	10	5	**0.007** (0.003)	0.012 (0.003)	0.020 (0.003)	0.015 (0.003)	0.014 (0.004)	0.013 (0.004)	0.013 (0.003)	0.020 (0.003)

Table S.2: Median and IQR (in parenthesis) of ISEs of eight estimators for estimating the 1st eigenfunction. The results are based on 200 replications.

Case	n	m	SNR	FACEs	FACEs(1-Stage)	TPRS	TPRS(50)	TPRS(97)	*fpca.sc*	MLE	*loc*
Case 1	100	5	2	0.038 (0.039)	0.050 (0.059)	0.042 (0.048)	0.067 (0.078)	0.075 (0.083)	0.071 (0.092)	0.050 (0.064)	**0.017** (0.017)
	400	5	2	0.013 (0.011)	0.019 (0.016)	0.015 (0.014)	0.025 (0.024)	0.029 (0.024)	0.025 (0.024)	**0.012** (0.012)	0.018 (0.016)
	100	10	2	**0.020** (0.026)	0.037 (0.043)	0.026 (0.025)	0.053 (0.056)	0.060 (0.058)	0.058 (0.062)	0.029 (0.034)	0.025 (0.025)
	400	10	2	0.008 (0.009)	0.012 (0.012)	0.009 (0.007)	0.015 (0.015)	0.017 (0.016)	0.016 (0.016)	**0.005** (0.007)	0.010 (0.008)
	100	5	5	0.025 (0.024)	0.045 (0.051)	0.040 (0.041)	0.062 (0.074)	0.066 (0.080)	0.064 (0.080)	0.032 (0.046)	**0.016** (0.020)
	400	5	5	**0.009** (0.010)	0.018 (0.017)	0.013 (0.010)	0.022 (0.022)	0.025 (0.024)	0.023 (0.024)	**0.009** (0.010)	0.018 (0.016)
	100	10	5	**0.014** (0.017)	0.032 (0.046)	0.024 (0.025)	0.046 (0.051)	0.053 (0.061)	0.052 (0.060)	0.025 (0.028)	0.021 (0.025)
	400	10	5	0.005 (0.006)	0.010 (0.012)	0.009 (0.007)	0.013 (0.015)	0.015 (0.016)	0.014 (0.015)	**0.004** (0.004)	0.008 (0.008)
Case 2	100	5	2	0.436 (0.748)	**0.359** (0.549)	0.554 (0.916)	0.675 (1.024)	0.666 (0.940)	0.689 (0.981)	0.869 (0.959)	0.515 (0.781)
	400	5	2	0.329 (0.556)	0.277 (0.500)	0.434 (0.698)	0.424 (0.699)	0.457 (0.684)	0.464 (0.730)	0.398 (0.718)	**0.209** (0.455)
	100	10	2	0.437 (0.610)	**0.393** (0.683)	0.513 (0.709)	0.597 (0.706)	0.621 (0.676)	0.645 (0.713)	0.578 (0.696)	0.408 (0.638)
	400	10	2	0.205 (0.517)	0.238 (0.517)	0.236 (0.491)	0.261 (0.513)	0.283 (0.513)	0.272 (0.516)	0.217 (0.457)	**0.154** (0.333)
	100	5	5	0.400 (0.701)	**0.366** (0.813)	0.592 (0.720)	0.609 (0.724)	0.674 (0.743)	0.710 (0.777)	0.786 (0.859)	0.452 (0.701)
	400	5	5	0.225 (0.496)	0.240 (0.495)	0.329 (0.477)	0.353 (0.524)	0.381 (0.507)	0.396 (0.644)	0.280 (0.604)	**0.167** (0.271)
	100	10	5	0.390 (0.643)	0.409 (0.710)	0.499 (0.735)	0.527 (0.767)	0.588 (0.794)	0.593 (0.828)	0.468 (0.668)	**0.362** (0.666)
	400	10	5	0.161 (0.327)	0.157 (0.375)	0.209 (0.323)	0.211 (0.326)	0.239 (0.321)	0.248 (0.327)	0.147 (0.298)	**0.095** (0.210)

Table S.3: Median and IQR (in parenthesis) of ISEs of eight estimators for estimating the 2nd eigenfunction. The results are based on 200 replications.

Case	n	m	SNR	FACEs	FACEs(1-Stage)	TPRS	TPRS(50)	TPRS(97)	*fpca.sc*	MLE	*loc*
Case 1	100	5	2	**0.179** (0.300)	0.387 (0.521)	1.452 (0.783)	0.339 (0.467)	0.343 (0.466)	0.304 (0.427)	0.185 (0.249)	0.450 (0.573)
	400	5	2	0.037 (0.037)	0.060 (0.065)	1.623 (0.468)	0.074 (0.067)	0.087 (0.077)	0.079 (0.071)	**0.036** (0.034)	0.146 (0.127)
	100	10	2	**0.069** (0.079)	0.128 (0.137)	1.505 (0.583)	0.127 (0.148)	0.146 (0.151)	0.141 (0.147)	0.070 (0.089)	0.133 (0.136)
	400	10	2	0.017 (0.021)	0.030 (0.036)	1.738 (0.323)	0.033 (0.035)	0.038 (0.036)	0.037 (0.036)	**0.015** (0.014)	0.073 (0.047)
	100	5	5	**0.086** (0.117)	0.196 (0.321)	1.449 (0.647)	0.237 (0.347)	0.256 (0.339)	0.218 (0.258)	0.096 (0.111)	0.362 (0.505)
	400	5	5	**0.020** (0.024)	0.042 (0.054)	1.675 (0.419)	0.059 (0.065)	0.065 (0.070)	0.061 (0.063)	0.021 (0.022)	0.124 (0.107)
	100	10	5	**0.043** (0.058)	0.091 (0.115)	1.560 (0.475)	0.094 (0.114)	0.105 (0.121)	0.101 (0.114)	0.050 (0.051)	0.107 (0.120)
	400	10	5	0.011 (0.012)	0.023 (0.025)	1.742 (0.309)	0.030 (0.025)	0.032 (0.029)	0.032 (0.030)	**0.009** (0.009)	0.070 (0.036)
Case 2	100	5	2	0.700 (0.856)	**0.529** (0.749)	0.883 (0.967)	1.011 (0.968)	0.966 (0.908)	1.050 (0.892)	1.238 (0.862)	0.865 (0.959)
	400	5	2	0.543 (0.823)	0.467 (0.783)	0.701 (0.946)	0.769 (0.936)	0.785 (0.976)	0.838 (0.962)	0.828 (0.782)	**0.450** (0.686)
	100	10	2	**0.640** (0.985)	0.684 (0.871)	0.779 (0.887)	0.862 (0.924)	0.959 (0.956)	0.969 (0.960)	0.971 (0.941)	0.729 (0.860)
	400	10	2	0.374 (0.658)	0.439 (0.612)	0.438 (0.785)	0.466 (0.738)	0.493 (0.706)	0.508 (0.760)	0.374 (0.583)	**0.271** (0.424)
	100	5	5	0.687 (0.951)	**0.600** (0.939)	0.826 (0.901)	0.889 (0.895)	1.012 (0.892)	1.060 (0.901)	1.101 (0.900)	0.815 (0.935)
	400	5	5	0.449 (0.676)	0.424 (0.627)	0.587 (0.704)	0.642 (0.785)	0.683 (0.809)	0.706 (0.841)	0.624 (0.934)	**0.364** (0.425)
	100	10	5	**0.701** (0.963)	0.704 (0.929)	0.876 (0.986)	0.993 (0.944)	0.998 (0.976)	1.035 (0.906)	0.881 (0.878)	0.723 (0.861)
	400	10	5	0.257 (0.446)	0.310 (0.487)	0.340 (0.492)	0.365 (0.589)	0.375 (0.576)	0.384 (0.579)	0.264 (0.437)	**0.203** (0.288)

Table S.4: Median and IQR (in parenthesis) of ISEs of eight estimators for estimating the 3rd eigenfunction. The results are based on 200 replications.

Case	n	m	SNR	FACEs	FACEs(1-Stage)	TPRS	TPRS(50)	TPRS(97)	*fpca.sc*	MLE	*loc*
Case 1	100	5	2	0.352 (0.455)	0.640 (0.707)	1.104 (0.998)	0.679 (0.828)	0.716 (0.689)	0.663 (0.670)	**0.339** (0.462)	0.799 (1.020)
	400	5	2	0.069 (0.061)	0.115 (0.099)	1.527 (0.553)	0.139 (0.121)	0.179 (0.136)	0.163 (0.114)	**0.057** (0.058)	0.229 (0.248)
	100	10	2	**0.110** (0.124)	0.193 (0.203)	1.425 (0.784)	0.190 (0.161)	0.238 (0.193)	0.226 (0.165)	0.117 (0.120)	0.200 (0.251)
	400	10	2	0.035 (0.040)	0.058 (0.044)	1.740 (0.355)	0.054 (0.042)	0.066 (0.043)	0.060 (0.044)	**0.021** (0.017)	0.078 (0.070)
	100	5	5	**0.156** (0.160)	0.435 (0.612)	1.177 (0.813)	0.497 (0.494)	0.511 (0.536)	0.470 (0.466)	**0.156** (0.164)	0.715 (0.827)
	400	5	5	0.036 (0.029)	0.094 (0.071)	1.558 (0.505)	0.099 (0.085)	0.132 (0.094)	0.119 (0.089)	**0.027** (0.025)	0.197 (0.194)
	100	10	5	**0.054** (0.066)	0.134 (0.149)	1.489 (0.629)	0.128 (0.126)	0.160 (0.164)	0.150 (0.143)	0.056 (0.061)	0.148 (0.188)
	400	10	5	0.014 (0.013)	0.041 (0.033)	1.723 (0.372)	0.040 (0.030)	0.049 (0.031)	0.046 (0.030)	**0.010** (0.009)	0.067 (0.052)
Case 2	100	5	2	0.610 (0.900)	**0.433** (0.738)	0.781 (0.992)	0.961 (0.842)	0.966 (0.824)	1.027 (0.823)	1.177 (0.852)	0.979 (0.827)
	400	5	2	0.363 (0.506)	**0.317** (0.431)	0.511 (0.728)	0.626 (0.793)	0.711 (0.804)	0.760 (0.795)	0.759 (0.795)	0.408 (0.489)
	100	10	2	0.663 (0.952)	**0.580** (0.745)	0.721 (0.917)	0.963 (0.897)	1.070 (0.896)	1.111 (0.869)	1.093 (0.907)	0.744 (0.826)
	400	10	2	0.308 (0.471)	0.331 (0.507)	0.319 (0.491)	0.421 (0.604)	0.495 (0.652)	0.505 (0.704)	0.392 (0.598)	**0.279** (0.242)
	100	5	5	0.449 (0.894)	**0.409** (0.718)	0.698 (1.010)	0.874 (0.957)	1.147 (0.931)	1.222 (0.972)	1.229 (0.757)	0.816 (0.910)
	400	5	5	**0.321** (0.582)	0.341 (0.463)	0.425 (0.646)	0.547 (0.682)	0.627 (0.681)	0.709 (0.698)	0.626 (0.725)	0.372 (0.362)
	100	10	5	0.812 (0.898)	**0.625** (0.915)	0.791 (0.858)	0.951 (0.776)	1.060 (0.776)	1.096 (0.789)	1.121 (0.846)	0.788 (0.823)
	400	10	5	**0.193** (0.252)	0.231 (0.371)	0.267 (0.375)	0.325 (0.423)	0.354 (0.436)	0.362 (0.455)	0.257 (0.300)	0.219 (0.199)

Table S.5: 100× Median and IQR (in parenthesis) of SEs of eight estimators for estimating the 1st eigenvalue. The results are based on 200 replications.

Case	n	m	SNR	FACEs	FACEs(1-Stage)	TPRS	TPRS(50)	TPRS(97)	*fpca.sc*	MLE	*loc*
Case 1	100	5	2	**1.736** (5.183)	2.030 (5.342)	2.914 (6.793)	2.647 (6.376)	2.306 (6.328)	2.335 (6.261)	3.877 (9.450)	5.749 (21.780)
	400	5	2	**0.501** (1.213)	0.551 (1.516)	0.624 (1.742)	0.599 (1.450)	0.613 (1.483)	0.603 (1.510)	4.911 (6.375)	11.312 (6.953)
	100	10	2	**1.212** (3.228)	2.206 (4.500)	1.772 (5.345)	1.520 (4.691)	1.554 (4.488)	1.486 (4.460)	4.009 (9.774)	4.366 (8.535)
	400	10	2	**0.258** (0.700)	0.314 (0.788)	0.290 (0.792)	0.301 (0.799)	0.286 (0.825)	0.284 (0.845)	4.840 (4.729)	8.184 (4.808)
	100	5	5	2.087 (4.322)	1.699 (4.230)	1.945 (5.038)	1.516 (4.750)	1.460 (4.605)	**1.463** (4.701)	2.708 (7.754)	5.927 (23.788)
	400	5	5	**0.370** (0.855)	0.410 (1.316)	0.602 (1.434)	0.581 (1.146)	0.524 (1.113)	0.540 (1.165)	4.783 (5.177)	11.382 (5.761)
	100	10	5	1.387 (3.910)	**1.291** (4.211)	1.582 (4.156)	1.335 (3.815)	1.347 (3.876)	1.337 (3.598)	4.295 (8.905)	4.576 (7.862)
	400	10	5	**0.192** (0.534)	0.514 (1.149)	0.311 (0.721)	0.302 (0.679)	0.306 (0.696)	0.308 (0.690)	4.888 (4.324)	8.222 (5.181)
Case 2	100	5	2	0.265 (0.716)	0.351 (0.715)	0.567 (1.193)	0.774 (1.425)	0.859 (1.659)	0.816 (1.530)	1.359 (1.865)	**0.211** (0.542)
	400	5	2	0.071 (0.185)	0.117 (0.281)	0.104 (0.312)	0.140 (0.407)	0.178 (0.440)	0.156 (0.435)	0.180 (0.281)	**0.047** (0.098)
	100	10	2	0.115 (0.269)	0.190 (0.534)	0.241 (0.556)	0.349 (0.663)	0.426 (0.808)	0.417 (0.786)	0.307 (0.601)	**0.084** (0.273)
	400	10	2	0.032 (0.068)	0.051 (0.143)	0.046 (0.092)	0.055 (0.104)	0.059 (0.123)	0.056 (0.113)	0.045 (0.093)	**0.026** (0.065)
	100	5	5	**0.127** (0.405)	0.214 (0.658)	0.331 (0.840)	0.435 (1.073)	0.555 (1.258)	0.537 (1.242)	0.783 (1.289)	0.134 (0.437)
	400	5	5	0.034 (0.105)	0.068 (0.170)	0.069 (0.202)	0.076 (0.239)	0.097 (0.273)	0.095 (0.254)	0.113 (0.229)	**0.033** (0.086)
	100	10	5	**0.063** (0.194)	0.161 (0.458)	0.154 (0.383)	0.181 (0.479)	0.230 (0.514)	0.225 (0.503)	0.207 (0.483)	0.074 (0.225)
	400	10	5	0.028 (0.080)	0.055 (0.133)	0.031 (0.081)	0.037 (0.098)	0.040 (0.109)	0.038 (0.106)	0.046 (0.091)	**0.023** (0.052)

Table S.6: 100× Median and IQR (in parenthesis) of SEs of eight estimators for estimating the 2nd eigenvalue. The results are based on 200 replications.

Case	n	m	SNR	FACEs	FACEs(1-Stage)	TPRS	TPRS(50)	TPRS(97)	*fpca.sc*	MLE	*loc*
Case 1	100	5	2	1.912 (2.943)	3.230 (5.565)	9.879 (6.997)	2.578 (4.115)	1.940 (3.333)	1.530 (3.118)	**0.486** (1.564)	9.088 (4.855)
	400	5	2	0.313 (0.889)	0.867 (1.936)	9.780 (3.503)	0.675 (1.248)	0.581 (1.095)	0.451 (0.927)	**0.146** (0.304)	11.891 (2.358)
	100	10	2	0.869 (1.573)	1.552 (2.927)	9.662 (4.534)	0.699 (1.730)	0.619 (1.673)	0.565 (1.554)	**0.261** (0.736)	7.351 (3.697)
	400	10	2	0.205 (0.444)	0.336 (0.929)	9.901 (2.740)	0.178 (0.418)	0.173 (0.487)	0.152 (0.435)	**0.096** (0.273)	8.810 (2.439)
	100	5	5	2.079 (2.585)	2.826 (5.186)	10.684 (5.510)	2.374 (4.433)	2.132 (3.606)	1.677 (3.346)	**0.455** (1.420)	9.634 (5.118)
	400	5	5	0.356 (0.769)	0.557 (1.523)	10.164 (3.484)	0.381 (0.973)	0.339 (0.945)	0.288 (0.762)	**0.100** (0.292)	12.017 (1.920)
	100	10	5	1.795 (1.835)	1.322 (2.811)	10.207 (4.581)	0.673 (1.509)	0.533 (1.356)	0.492 (1.327)	**0.242** (0.681)	7.831 (4.057)
	400	10	5	0.311 (0.441)	0.302 (0.595)	9.793 (1.964)	0.124 (0.380)	0.128 (0.323)	0.123 (0.295)	**0.055** (0.175)	9.106 (2.132)
Case 2	100	5	2	0.165 (0.333)	0.300 (0.766)	0.177 (0.447)	0.123 (0.397)	0.157 (0.430)	0.174 (0.430)	0.241 (0.596)	**0.140** (0.333)
	400	5	2	**0.021** (0.060)	0.040 (0.090)	0.027 (0.065)	0.024 (0.070)	0.028 (0.077)	0.027 (0.081)	0.035 (0.077)	0.098 (0.196)
	100	10	2	0.063 (0.158)	0.077 (0.172)	0.061 (0.146)	0.057 (0.160)	0.064 (0.200)	0.067 (0.207)	**0.046** (0.144)	0.091 (0.212)
	400	10	2	0.015 (0.035)	0.018 (0.050)	**0.012** (0.050)	0.013 (0.054)	0.016 (0.054)	0.014 (0.054)	0.015 (0.040)	0.056 (0.121)
	100	5	5	**0.079** (0.252)	0.180 (0.461)	0.128 (0.316)	0.090 (0.254)	0.089 (0.278)	0.089 (0.270)	0.136 (0.302)	0.154 (0.367)
	400	5	5	**0.016** (0.042)	0.031 (0.074)	0.029 (0.071)	0.027 (0.075)	0.027 (0.075)	0.029 (0.069)	0.028 (0.069)	0.110 (0.216)
	100	10	5	0.041 (0.102)	0.058 (0.109)	0.043 (0.115)	0.046 (0.118)	0.048 (0.126)	0.043 (0.125)	**0.038** (0.098)	0.115 (0.211)
	400	10	5	0.012 (0.037)	0.016 (0.041)	**0.010** (0.034)	0.012 (0.031)	0.012 (0.030)	0.012 (0.033)	0.011 (0.036)	0.068 (0.126)

Table S.7: 100× Median and IQR (in parenthesis) of SEs of eight estimators for estimating the 3rd eigenvalue. The results are based on 200 replications.

Case	n	m	SNR	FACEs	FACEs(1-Stage)	TPRS	TPRS(50)	TPRS(97)	*fpca.sc*	MLE	*loc*
Case 1	100	5	2	**0.167** (0.491)	0.791 (1.532)	3.147 (2.115)	0.717 (1.254)	0.466 (1.074)	0.500 (1.095)	0.224 (0.711)	2.521 (1.915)
	400	5	2	**0.077** (0.197)	0.154 (0.367)	2.778 (1.909)	0.164 (0.464)	0.155 (0.420)	0.157 (0.406)	0.088 (0.218)	2.986 (0.959)
	100	10	2	0.115 (0.325)	0.406 (0.834)	2.964 (1.778)	0.329 (0.679)	0.262 (0.531)	0.285 (0.552)	**0.114** (0.371)	2.337 (1.448)
	400	10	2	0.037 (0.084)	0.082 (0.187)	3.113 (1.452)	0.107 (0.216)	0.093 (0.196)	0.094 (0.186)	**0.028** (0.087)	2.581 (0.833)
	100	5	5	0.274 (0.537)	0.759 (1.449)	3.237 (2.045)	0.615 (1.031)	0.438 (0.949)	0.453 (0.914)	**0.192** (0.518)	2.646 (1.684)
	400	5	5	**0.034** (0.084)	0.113 (0.269)	3.049 (1.690)	0.132 (0.340)	0.127 (0.269)	0.122 (0.290)	0.038 (0.086)	3.066 (0.808)
	100	10	5	0.130 (0.288)	0.308 (0.613)	3.059 (1.669)	0.216 (0.565)	0.191 (0.470)	0.193 (0.427)	**0.093** (0.224)	2.416 (1.112)
	400	10	5	**0.019** (0.054)	0.043 (0.128)	2.960 (1.349)	0.055 (0.151)	0.040 (0.123)	0.038 (0.116)	0.021 (0.063)	2.680 (0.590)
Case 2	100	5	2	0.418 (1.067)	0.644 (1.441)	0.407 (0.826)	0.200 (0.517)	0.168 (0.539)	0.184 (0.501)	**0.085** (0.230)	0.370 (0.474)
	400	5	2	0.029 (0.083)	0.060 (0.150)	0.056 (0.138)	0.039 (0.083)	0.033 (0.076)	0.030 (0.067)	**0.016** (0.039)	0.258 (0.311)
	100	10	2	0.072 (0.154)	0.146 (0.299)	0.097 (0.188)	0.047 (0.122)	0.038 (0.109)	0.035 (0.100)	**0.019** (0.065)	0.227 (0.294)
	400	10	2	0.012 (0.032)	0.014 (0.034)	0.018 (0.048)	0.014 (0.035)	0.013 (0.035)	0.013 (0.037)	**0.011** (0.027)	0.162 (0.151)
	100	5	5	0.204 (0.547)	0.441 (1.065)	0.257 (0.478)	0.143 (0.328)	0.095 (0.259)	0.092 (0.294)	**0.060** (0.203)	0.380 (0.443)
	400	5	5	**0.013** (0.041)	0.029 (0.079)	0.039 (0.090)	0.028 (0.068)	0.022 (0.050)	0.019 (0.051)	0.017 (0.048)	0.224 (0.236)
	100	10	5	0.044 (0.102)	0.081 (0.215)	0.067 (0.149)	0.039 (0.090)	0.026 (0.071)	0.026 (0.072)	**0.023** (0.054)	0.217 (0.251)
	400	10	5	**0.010** (0.028)	0.016 (0.033)	0.013 (0.037)	0.012 (0.031)	0.013 (0.030)	0.014 (0.029)	**0.010** (0.025)	0.154 (0.159)

Table S.8: Median and IQR (in parenthesis) of computation times (in seconds) of eight estimators for estimating the covariance functions on a desktop with a 2.3 GHz CPU and 8 GB of RAM. The results are based on 200 replications.

Case	n	m	SNR	FACEs	FACEs(1-Stage)	TPRS	TPRS(50)	TPRS(97)	*fpca.sc*	MLE	*loc*
Case 1	100	5	2	14.1 (1.1)	5.6 (0.2)	4.1 (1.2)	6.1 (0.2)	9.8 (0.4)	2.4 (1.2)	141.7 (52.7)	477.3 (43.8)
	400	5	2	56.6 (12.3)	15.3 (0.5)	8.5 (2.9)	11.1 (0.7)	79.8 (44.2)	11.0 (7.1)	615.1 (209.6)	1304.8 (239.0)
	100	10	2	19.3 (1.0)	5.3 (0.3)	7.5 (1.4)	10.5 (1.1)	37.8 (13.4)	12.2 (4.4)	153.1 (41.7)	1680.5 (258.9)
	400	10	2	79.6 (2.3)	21.5 (6.4)	23.3 (4.0)	34.7 (8.4)	200.6 (68.5)	55.7 (17.5)	652.8 (149.3)	772.8 (31.5)
	100	5	5	15.6 (0.2)	4.7 (0.2)	4.6 (0.2)	5.6 (0.2)	16.7 (5.3)	2.9 (0.3)	174.2 (26.0)	462.5 (83.8)
	400	5	5	66.4 (3.9)	21.5 (2.3)	10.0 (2.0)	14.3 (1.9)	55.0 (11.6)	14.1 (4.5)	767.3 (216.4)	2462.8 (387.2)
	100	10	5	16.0 (1.1)	6.2 (0.5)	6.9 (1.5)	12.6 (1.6)	36.6 (6.0)	10.9 (3.3)	143.9 (46.5)	2781.0 (426.8)
	400	10	5	81.0 (1.7)	21.0 (1.6)	23.6 (4.9)	33.8 (3.1)	217.6 (75.7)	55.6 (17.4)	758.3 (137.2)	486.0 (36.7)
Case 2	100	5	2	16.7 (3.9)	5.8 (0.3)	4.1 (0.3)	6.3 (0.4)	17.1 (6.6)	2.3 (0.3)	166.6 (28.7)	1035.5 (112.5)
	400	5	2	50.0 (17.5)	16.3 (0.8)	8.9 (2.4)	11.4 (0.7)	81.5 (39.4)	11.4 (4.1)	907.6 (262.2)	2704.5 (1303.5)
	100	10	2	21.4 (1.4)	6.2 (0.7)	6.2 (0.6)	12.3 (2.0)	37.0 (12.5)	9.6 (2.1)	150.3 (23.1)	2302.8 (461.4)
	400	10	2	70.3 (1.7)	20.7 (2.1)	19.5 (3.7)	31.4 (3.9)	186.5 (64.9)	42.7 (13.7)	701.2 (117.8)	362.8 (63.3)
	100	5	5	15.2 (0.1)	3.7 (0.2)	4.1 (0.2)	4.5 (0.3)	9.7 (1.6)	2.4 (0.2)	179.8 (17.8)	739.4 (60.1)
	400	5	5	57.3 (2.9)	19.4 (0.6)	9.0 (0.6)	12.7 (0.8)	19.2 (5.1)	13.4 (1.7)	835.1 (97.7)	2041.2 (981.4)
	100	10	5	14.8 (0.4)	5.6 (0.4)	6.9 (0.3)	11.3 (1.1)	28.9 (3.2)	11.3 (1.1)	172.0 (13.0)	2719.9 (1005.6)
	400	10	5	58.5 (1.3)	22.9 (0.6)	22.1 (5.3)	35.1 (2.2)	56.5 (7.4)	52.9 (17.8)	772.8 (210.8)	1532.4 (247.8)

last two decades Pampas has undergone many economic and social developments. The study contains 197 children with anthropomorphic measurements taken from birth. Here we focus on the length curves from birth to 1 year. Each child has 10 to 32 measurements of length, with 4320 data points in total. Figure S.1 displays the sample length trajectories. We apply the proposed method to the data. The estimated population mean is plotted in Figure S.1 as a dashed line. The estimated mean curve is generally increasing with age, which is expected. In Figure S.2, we plot the estimated variance and correlation functions. The estimated variance function is increasing as a function of age. The estimated correlation function is shown as a heat map. Each point in the heat map represents the estimated correlation between two days and the color corresponds to the correlation values with red indicating higher correlation and blue indicating lower correlation. The diagonal is dark red indicating perfect correlation, while the minimum correlation is about 0.2. Given the estimated covariance function, using the framework described in Section 4, we predict each child's length trajectories. Figure S.3 displays for 4 children the predicted length trajectories with point-wise confidence bands. We randomly sample varying numbers of observations to see the effect of the number of observations on the prediction. We see that the point-wise confidence intervals are generally narrower with a larger number of observations, which is also as expected.

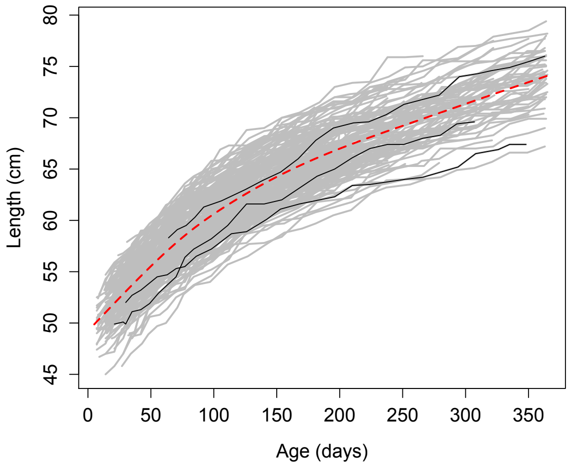

Figure S.1: Length trajectories of about 200 children from birth to 1 year old. The estimated population mean is the dashed red line.

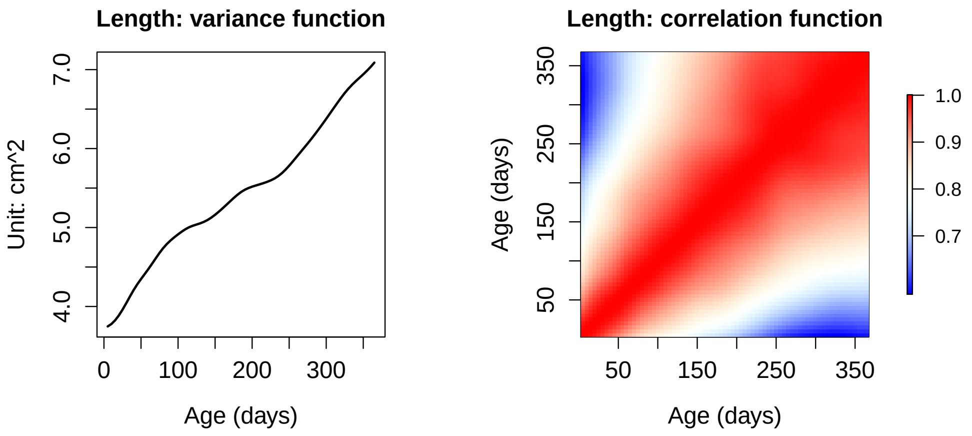

Figure S.2: Estimated variance function (left panel) and correlation function (right panel) for the length of children from birth to 1 year old.

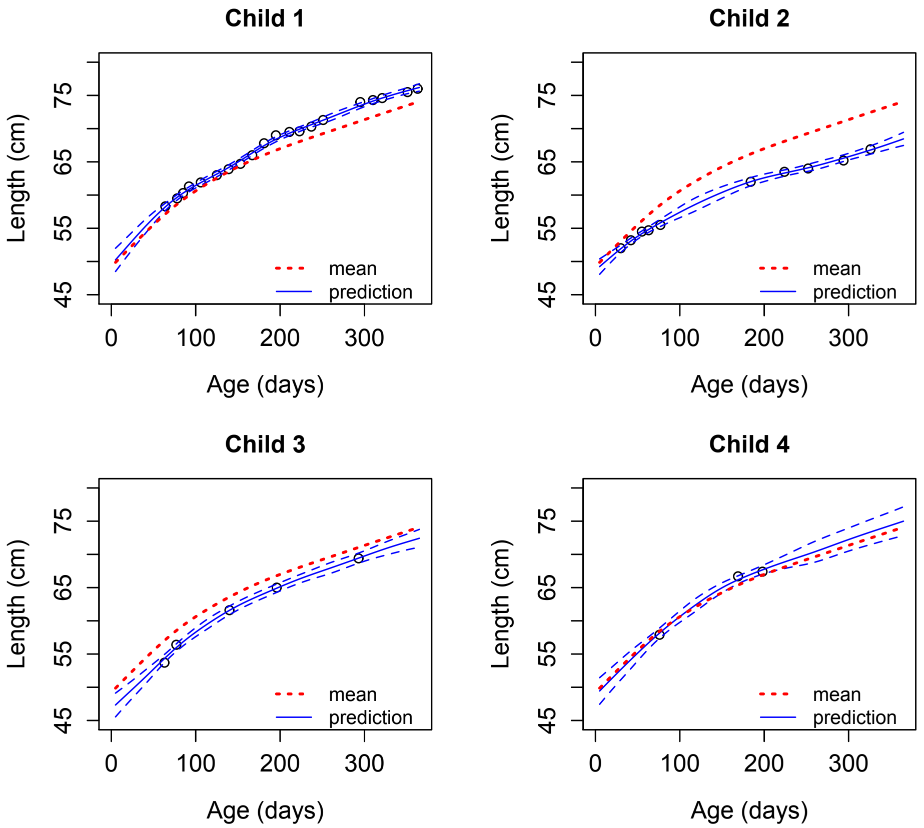

Figure S.3: Predicted child-specific length trajectories from birth to 1 year old and associated 95% confidence bands for 4 children. The estimated population mean is the dotted red line.

References

Isserlis, L. (1918, November). On a formula for the product-moment coefficient of any order of a normal frequency distribution in any number of variables. *Biometrika 12*(1-2), 134–139.
